# An infection-responsive multifunctional hydrogel enables potent activity against methicillin-resistant *Staphylococcus aureus* and promotes wound healing

**DOI:** 10.1016/j.mtbio.2026.103494

**Published:** 2026-07-25

**Authors:** Chuanliang Fu, Wenjing Zhang, Renyuan Wang, Runyi Cao, Zhengjie Chen, Peilin Wang, Ying Peng, Yilin Huo, Yi Hu, Zun Ren, Hao Zhang, Yersen Mulat, Zhengjie Luo, Yahui Dai, Min Zhou, Haodong Lin

**Affiliations:** a Trauma Center, Shanghai General Hospital, Shanghai Jiao Tong University School of Medicine, Shanghai, 201620, China; b Precision Research Center for Refractory Diseases, Shanghai General Hospital, Shanghai Jiao Tong University School of Medicine, Shanghai, 201620, China; c Key Laboratory for Ultrafine Materials of Ministry of Education, Frontiers Science Center for Materiobiology and Dynamic Chemistry, Shanghai Frontiers Science Center of Optogenetic Techniques for Cell Metabolism, Research Center for Biomedical Materials of Ministry of Education, School of Materials Science and Engineering, East China University of Science and Technology, Shanghai, 200237, China

**Keywords:** Poly(2-oxazoline), Wound healing, MRSA, ZIF-8, Host defense peptide

## Abstract

Infected wounds remain a significant clinical challenge due to bacterial resistance and impaired healing. Therefore, developing effective antibacterial agents and precise delivery systems is crucial for rapid wound repair. To address this, we constructed zeolitic imidazolate framework-8 (ZIF-8) nanoparticles loaded with the host defense peptide-mimicking glycine-poly(2-oxazoline) (Gly-POX) and incorporated them into methacrylated gelatin (GelMA) to prepare the Gel-P@Z nanocomposite hydrogel. The hydrogel integrates the following core design elements: the pH-responsive degradation of ZIF-8 enables targeted drug release within the infected microenvironment; Gly-POX, mimicking the structure of host defense peptides, exerts membrane-disruptive antibacterial activity against MRSA, which possesses a negatively charged cell membrane, through its positively charged side chains; and the GelMA hydrogel provides a three-dimensional extracellular matrix-like scaffold that supports cell adhesion and proliferation. Gel-P@Z exhibited a slow and sustained release of Gly-POX and achieved >99% antibacterial efficacy against drug-resistant bacteria without toxicity. Moreover, Gel-P@Z promoted macrophage polarization from M1 to M2 phenotype and enhanced efferocytosis, while also facilitating fibroblast migration and inducing contraction in *ex vivo* fascia explants. In a murine full-thickness MRSA-infected wound model, Gel-P@Z effectively cleared bacteria, modulated the inflammatory microenvironment, and promoted both angiogenesis and collagen deposition. RNA-seq analysis revealed that Gel-P@Z accelerated healing via upregulation of the TGF-β signaling pathway, driving fibroblast-to-myofibroblast transition and promoting tissue fibrosis. This work not only proposes a novel strategy for antibacterial polymer delivery but also offers a promising solution for the management of infected wounds.

## Highlights

• An infection-responsive Gel-P@Z hydrogel was developed for MRSA-infected wound repair.
• Gel-P@Z promotes M2 macrophage polarization and enhances efferocytosis.
• Gel-P@Z promotes fibroblast migration, extracellular matrix remodeling and fascia contraction.
• Gel-P@Z accelerates wound healing via TGF-β/SMAD activation.

## Graphical abstract

**Figure:**
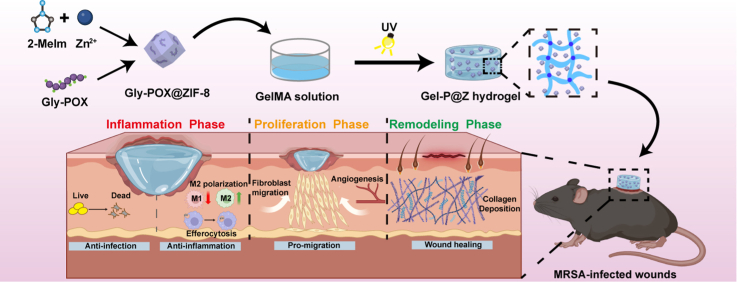

## 1 Introduction

Bacterial infections can disrupt wound healing, causing delays and making treatment difficult. Infected wounds are slow to heal, showing persistent inflammation, poor extracellular matrix remodeling, impaired cell function, and hindered tissue regeneration [1]. *Staphylococcus aureus* is one of the most frequently isolated bacteria in infected wounds, and the rapid emergence of drug-resistant bacteria, such as methicillin-resistant *Staphylococcus aureus* (MRSA), presents a significant challenge for clinical management [2]. Therefore, the development of effective therapeutics for the treatment of *S. aureus*-infected wounds, especially drug-resistant *S. aureus*, is urgently needed.

Hydrogels are promising for tissue engineering due to their biocompatibility and porous structure [3]. However, traditional hydrogel dressings mainly serve as passive barriers and moisture retainers, falling short for infected wound repair. Antibiotics are the primary treatment for such wounds, but incorporating them into dressings poses challenges: infections often recur due to antibiotic resistance, and healing is impeded by excessive inflammation and cell damage [4]. Recent advances in antibiotic-free wound healing biomaterials offer promising alternatives to traditional antibiotics for treating drug-resistant wound infections, such as multi-modal metal composite hydrogels, regulated bacterial cell death modalities, and multifunctional nanozyme-hydrogel dressings [[5], [6], [7]]. Antibiotic-free strategies have thus become a vital emerging approach to overcome the crisis of antibacterial resistance.

Zinc ions (Zn^2+^) are crucial trace elements in human physiology, known for their biocompatibility, affordability, and healing properties [8,9], including inflammation modulation, collagen formation, and angiogenesis enhancement. They promote macrophage transition to the anti-inflammatory M2 phenotype, reduce pro-inflammatory cytokines, and boost reparative factors [10,11]. Zn^2+^ also disrupts bacterial metabolism by affecting arginine synthesis and generating reactive oxygen species. [12,13]. However, directly applying free Zn^2+^ is challenging due to difficulties in maintaining effective concentrations without causing cellular toxicity. Thus, zeolitic imidazolate framework-8 (ZIF-8), a metal-organic framework composed of Zn^2+^ and 2-methylimidazole (2-MeIm), is particularly advantageous due to its large surface area, efficient loading of therapeutic agents, pH-responsive biodegradation, and good biocompatibility [14,15]. These properties enable sustained delivery while cooperating synergistic agents.

Due to the insufficient antibacterial activity of ZIF-8 and the potential issue of bacterial antibiotic resistance, host defense peptides (HDPs) emerge as a prime candidate owing to their broad-spectrum antimicrobial activity and low propensity for inducing antimicrobial resistance [16,17]. However, the application of HDPs is limited due to inherent shortcomings of natural peptides such as poor structural stability, difficult and costly production [18]. Recently, we developed HDP-mimicking polymers glycine-poly(2-oxazoline) (Gly-POX) that displayed potent broad-spectrum antibacterial activities against drug-resistant bacteria, including MRSA, no susceptibility to antibacterial resistance, and superior stability [19,20]. Therefore, we designed a multifunctional strategy by loading antibacterial Gly-POX with ZIF-8 nanoparticles, hypothesizing that this approach could offer a promising solution for achieving immunomodulation, the inhibition of wound infections and the enhancement of cell migration. Beyond antibacterial activity and immunomodulation, recent studies have demonstrated that wound repair also relies on the active mobilization of the underlying fascia [21]. Rather than serving as a passive connective tissue, the fascia functions as a reservoir of reparative fibroblasts and extracellular matrix that rapidly mobilize toward the wound bed after injury, thereby contributing to wound closure and tissue regeneration [22]. These findings suggest that promoting fascia mobilization represents a promising yet largely unexplored strategy for enhancing wound healing. In addition, GelMA hydrogel has a porous structure, degradability, biocompatibility, and adjustable physicochemical properties, which can serve as an excellent matrix for the transport and delivery of wound repair nanoparticles [23]. The three-dimensional network of GelMA hydrogel provides a good environment for the controlled release of ions and drugs, supporting cell proliferation, migration, and differentiation [24].

**Scheme 1:**
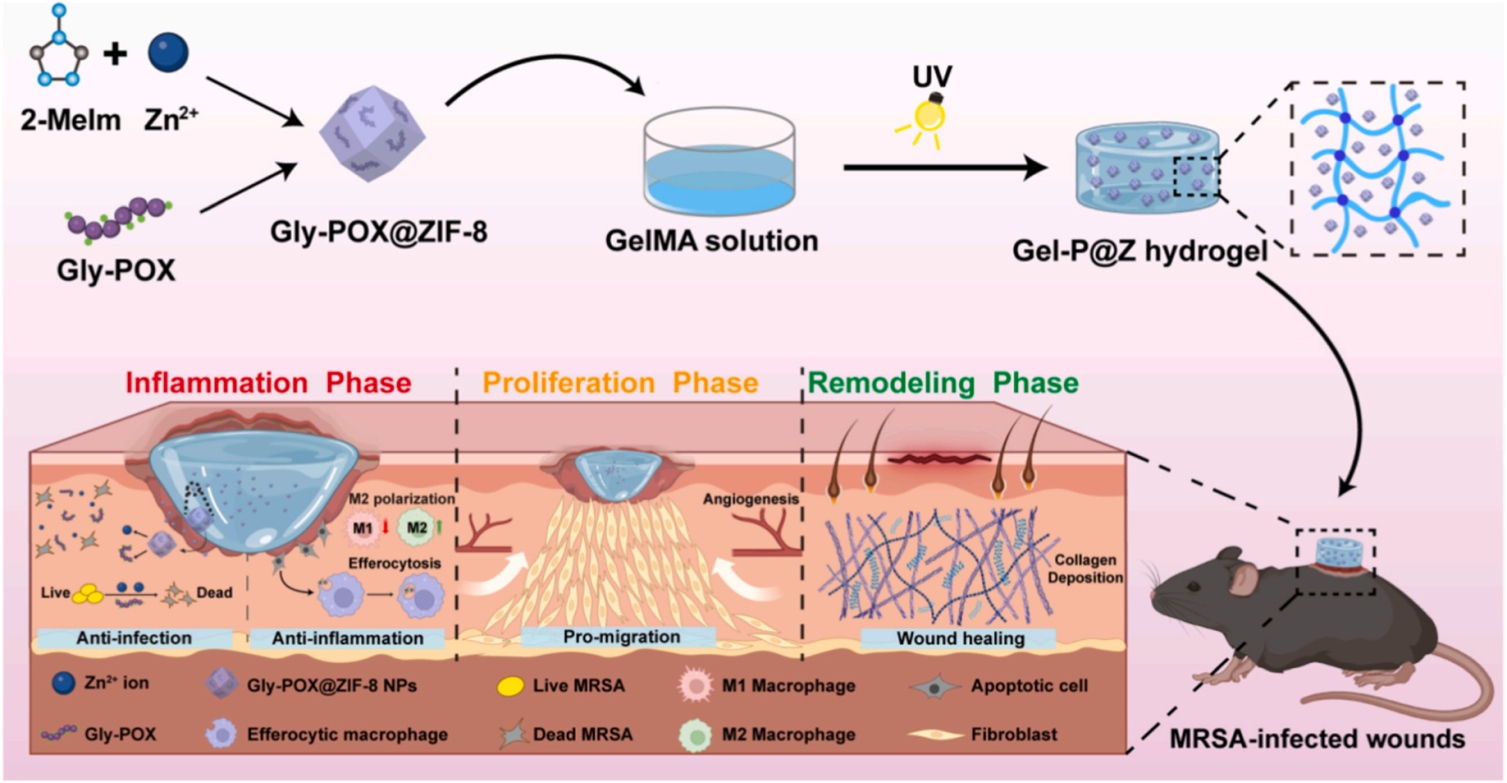
Schematic illustration of the fabrication of multi-functional hydrogel (Gel-P@Z) and treatment of methicillin-resistant *Staphylococcus aureus* (MRSA) infected wound by Gel-P@Z hydrogel with both antibacterial and pro-healing functions.

In this study, HDP-mimicking polymer Gly-POX was used as the antibacterial agent, and ZIF-8 was selected as a pH-responsive nanocarrier for its modification and Zn^2+^ reservoir for wound healing. The nanoparticles were incorporated into a GelMA hydrogel to fabricate a composite hydrogel (Gel-P@Z) with both anti-infection and pro-healing functions. The design of this hydrogel fully integrates the antibacterial advantages of HDP mimics, the acid-responsive controlled release properties of ZIF-8, and the three-dimensional scaffolding function of the GelMA hydrogel. We systematically evaluated the morphology, swelling behavior, drug release profile, antibacterial properties, and biocompatibility of the Gel-P@Z hydrogel *in vitro*. Additionally, we investigated its effects on macrophage polarization and efferocytosis, as well as fibroblast migration and fascial contraction. *In vivo*, the healing efficacy of Gel-P@Z was assessed using a murine model of MRSA-infected full-thickness skin wounds. Finally, transcriptomic analysis was employed to explore the potential mechanism by which Gel-P@Z promotes wound healing. Our findings demonstrate that Gel-P@Z accelerates infected wound healing through the core “antibacterial-anti-inflammatory-pro-migratory” mechanism, offering a novel delivery strategy based on antibacterial-mimetic polymers for the management of infected wounds (Scheme 1).

## 2 Results and discussion

### 2.1 The preparation and characterization of the nanocomposite hydrogel Gel-P@Z

**Fig. 1:**
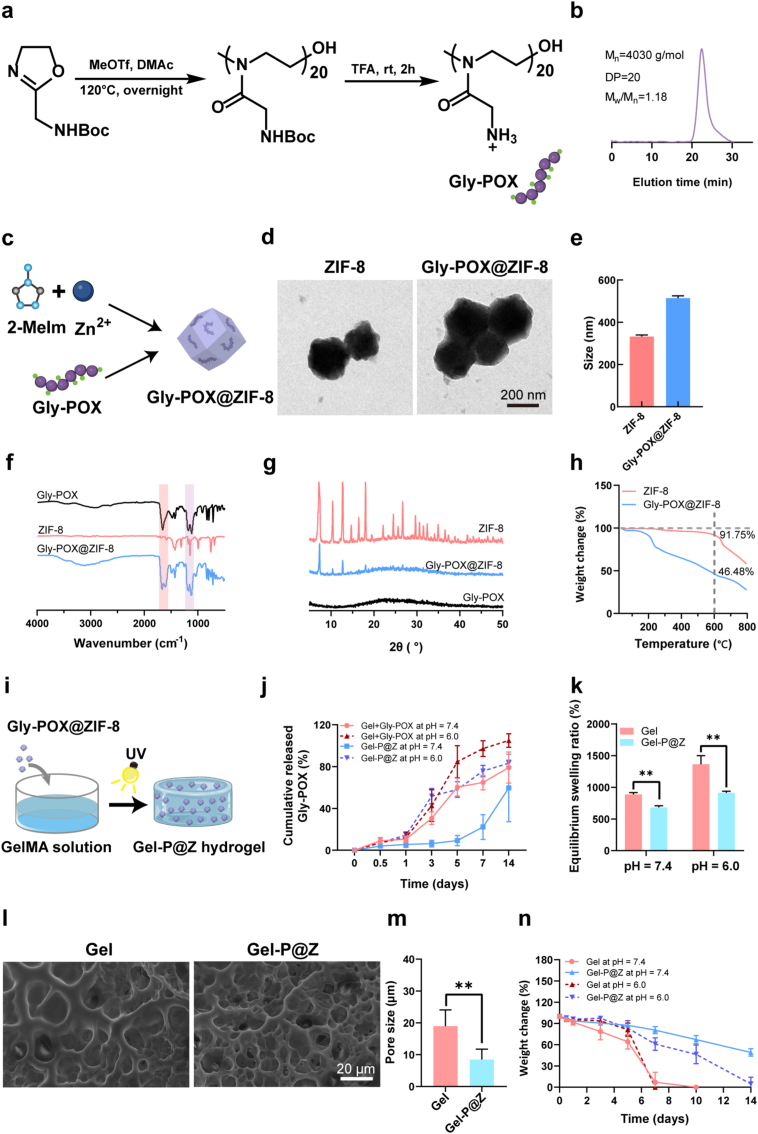
**Preparation of antibacterial poly(2-oxazoline) Gly-POX, nanocarrier ZIF-8 and nanocomposite hydrogels (Gel-P@Z)**. (a) Synthetic scheme of antibacterial poly(2-oxazoline) Gly-POX via cationic ring-opening polymerization using methyl trifluoromethanesulfonate (MeOTf) as the initiator and *N,N*-dimethylacetamide (DMAc) as the optimal solvent. (b) GPC trace of poly(2-oxazoline) at the side-chain protected stage. (c) Scheme of the preparation of Gly-POX-loaded ZIF-8 (Gly-POX@ZIF-8). (d) Representative TEM images displaying ZIF-8 and Gly-POX@ZIF-8 NPs. (e) DLS analysis of the size distribution of ZIF-8 and Gly-POX@ZIF-8. (f) FTIR spectra of ZIF-8, Gly-POX and Gly-POX@ZIF-8 NPs. (g) XRD analysis of ZIF-8, Gly-POX and Gly-POX@ZIF-8 NPs. (h) TGA curves of ZIF-8 and Gly-POX@ZIF-8 NPs. (i) Scheme of the preparation of Gel-P@Z. (j) Release behavior of Gly-POX from Gel-P@Z and Gel + Gly-POX at pH = 6.0 or 7.4 for 14 days (n = 3). (k) Equilibrium swelling ratio of Gel-P@Z hydrogels in PBS at pH = 6.0 or 7.4 after 48 h incubation (n = 3). (l) Morphology of the Gel and Gel-P@Z hydrogels using SEM analysis. (m) Pore sizes of hydrogels calculated from (l). (n) *In vitro* weight change profiles of the Gel and Gel-P@Z hydrogels in PBS at pH = 6.0 or 7.4 over a period of 14 days at rt (n = 3). Data are presented as mean ± SD. *p < 0.05, **p < 0.01; ns, not significant.

Antibacterial poly(2-oxazoline) was prepared from the cationic ring-opening polymerization of *N*-Boc-aminomethyl-2-oxazoline at 120°C using methyl trifluoromethanesulfonate (MeOTf) as the initiator and *N,N*-dimethylacetamide (DMAc) as the optimal solvent according to our previous study [19,20] (Fig. 1a). Poly(2-oxazoline) was obtained at 20 mer (DP = 20) and a narrow dispersity (*Đ* = 1.18) at NHBoc protected stage (Fig. 1b). Followed by removal of the *N*-Boc protecting group with trifluoroacetic acid (TFA), the final poly(2-oxazoline), named Gly-POX, was characterized by ^1^H NMR to confirm the controlled polymer structures (Fig. S1).

Then, the nanocarrier ZIF-8 with high loading efficiency was prepared through a solvent method as reported in the previous work [25] (Fig. 1c). To realize the antibacterial function, Gly-POX was loaded in ZIF-8 nanoparticles (NPs) by the impregnation method. We found that the mass ratio of Gly-POX to ZIF-8 was 1:1, and the resulting Gly-POX@ZIF-8 had the highest surface anchoring performance compared with 2:1 and 1:2, and the optimal synthesis conditions were determined. Therefore, all subsequent Gly-POX@ZIF-8 samples were synthesized using this ratio (Fig. S2). ZIF-8 and Gly-POX@ZIF-8 NPs exhibited the hexagonal shape (Fig. 1d). Dynamic light scattering (DLS) measurements indicated an average size of 514.0 nm for Gly-POX@ZIF-8 NPs larger than that of ZIF-8 NPs of 332.8 nm (Fig. 1e). Compared with ZIF-8 NPs, the zeta potentials of Gly-POX@ZIF-8 NPs did not change significantly (Fig. S3). The characteristic C=N stretching peak of ZIF-8 at ∼1580 cm^−1^ and the C=O stretching peak of Gly-POX at ∼1650 cm^−1^ showed obvious shifts in the spectrum of Gly-POX@ZIF-8, confirming the formation of coordination/hydrogen-bond interactions between Gly-POX and the ZIF-8 framework. The imidazole ring vibration peaks of ZIF-8 in the ∼1000–1300 cm^−1^ region also changed significantly, verifying successful composite construction (Fig. 1f). These spectral changes confirmed dual interfacial interactions: coordination interactions between polar functional groups of Gly-POX and unsaturated Zn^2+^ sites on the ZIF-8 framework, as well as hydrogen-bond interactions between Gly-POX functional groups and imidazole ligands of ZIF-8. Pure ZIF-8 displayed sharp diffraction peaks corresponding to its typical crystal structure, while Gly-POX was amorphous with only a broad diffuse peak. Gly-POX@ZIF-8 maintained the main diffraction peaks of ZIF-8, confirming retention of the ZIF-8 crystalline framework, with reduced peak intensity resulting from amorphous Gly-POX incorporation, verifying successful composite fabrication without disrupting the basic ZIF-8 crystal structure (Fig. 1g). The thermogravimetric analysis (TGA) results showed that pure ZIF-8 retained 91.75% weight at 600°C, while Gly-POX@ZIF-8 had a residual weight of only 46.48%.Based on the residual weight data, the approximate loading amount of Gly-POX was calculated to be 49.3 wt%, confirming successful incorporation of Gly-POX into the ZIF-8 matrix, accompanied by a reduction in overall thermal stability (Fig. 1h).

We used biocompatible and biodegradable gelatin as the matrix to construct the antibacterial and wound-healing hydrogel for treating MRSA-infected wounds. GelMA nanocomposite hydrogels (Gel-P@Z) were prepared by mixing GelMA within Gly-POX@ZIF-8 NPs followed by photo-crosslinking with UV light (Fig. 1i). The growth of microbes generates metabolic byproducts that may create a mildly acidic environment, while ZIF-8 functions as a framework material responsive to acidic conditions [20]. Under such acidic circumstances, the structure of ZIF-8 can disintegrate, which results in the release of the loaded drugs. Consequently, we examined the release characteristics of poly(2-oxazoline) from Gel-P@Z hydrogels in an acidic environment by assessing the acid-responsive release behavior of Gel-P@Z (Fig. 1j). The cumulative release of Gly-POX was monitored over 14 days at pH 7.4 and pH 6.0. Although the Gel + Gly-POX group and Gel-P@Z group exhibited similar continuous release trends, free Gly-POX displayed a distinctly faster release rate under the mildly acidic condition (pH 6.0), with a continuously rising cumulative release amount throughout the incubation period. In comparison, the Gel-P@Z composite hydrogel with Gly-POX loaded on ZIF-8 achieved slow, steady, and sustained Gly-POX release. Both systems showed obvious pH-responsive release behavior: a faster release rate was observed at pH 6.0 than at pH 7.4, as the acidic microenvironment weakens the coordination interaction between Gly-POX and ZIF-8 and accelerates matrix degradation. This finely regulated release behavior of Gel-P@Z effectively accelerates drug delivery appropriately under acidic conditions while maintaining prolonged antibacterial delivery, which well matches the physiological characteristics of infected wound microenvironments. Following a 48 h incubation in PBS at pH = 7.4, the equilibrium swelling ratio for the Gel-P@Z hydrogel was notably lower compared to that of the Gel hydrogel, which showed swelling ratios of 684.15% and 887.65%, respectively (Fig. 1k). Similarly, in PBS at pH 6.0, the equilibrium swelling ratio of the Gel-P@Z hydrogel remained notably lower than that of the Gel hydrogel, with swelling ratios of 913.6% and 1363.4%, respectively. Both groups showed increased swelling ratios at pH 6.0 relative to pH 7.4, demonstrating pH-responsive swelling behavior of the hydrogel matrix. The reduced swelling of Gel-P@Z benefits the structural integrity and sustained degradation of the hydrogel in the acidic wound microenvironment. The addition of ZIF-8 NPs and cationic poly(2-oxazoline) into Gel resulted in a more compact microstructure (Fig. 1l). The average pore sizes of lyophilized Gel and Gel-P@Z were 18.96 and 8.48 μm, respectively (Fig. 1m). The reduction of pore size and swelling ratio was attributed to the increased cross-linking density and the enhanced structural stability of Gel-P@Z. In addition, the addition of Gly-POX@ZIF-8 NPs showed no significant effects on the storage modulus of hydrogels (Fig. S4). In order to mimic the degradation behavior of Gel-P@Z hydrogels *in vivo*, the degradation profiles of the pure Gel and Gel-P@Z composite hydrogels were monitored via weight change measurements over 14 days at pH 7.4 and pH 6.0 (Fig. 1n). The pure Gel group exhibited rapid degradation under both conditions, especially at mildly acidic pH 6.0, with nearly complete weight loss by day 7. In contrast, the Gel-P@Z hydrogel displayed much slower, sustained degradation kinetics. At neutral pH 7.4, Gel-P@Z maintained a gradual, steady weight reduction over the entire 14-day period. At pH 6.0, Gel-P@Z showed accelerated yet still sustained degradation compared to pH 7.4, confirming its pH-responsive degradation behavior. The incorporation of Gly-POX@ZIF-8 effectively slowed bulk hydrogel degradation and enabled tunable, prolonged matrix breakdown, which supports long-term localized treatment in the mildly acidic wound microenvironment.

### 2.2 Antibacterial activity and biocompatibility of Gel-P@Z evaluation *in vitro* and *in vivo*

**Fig. 2:**
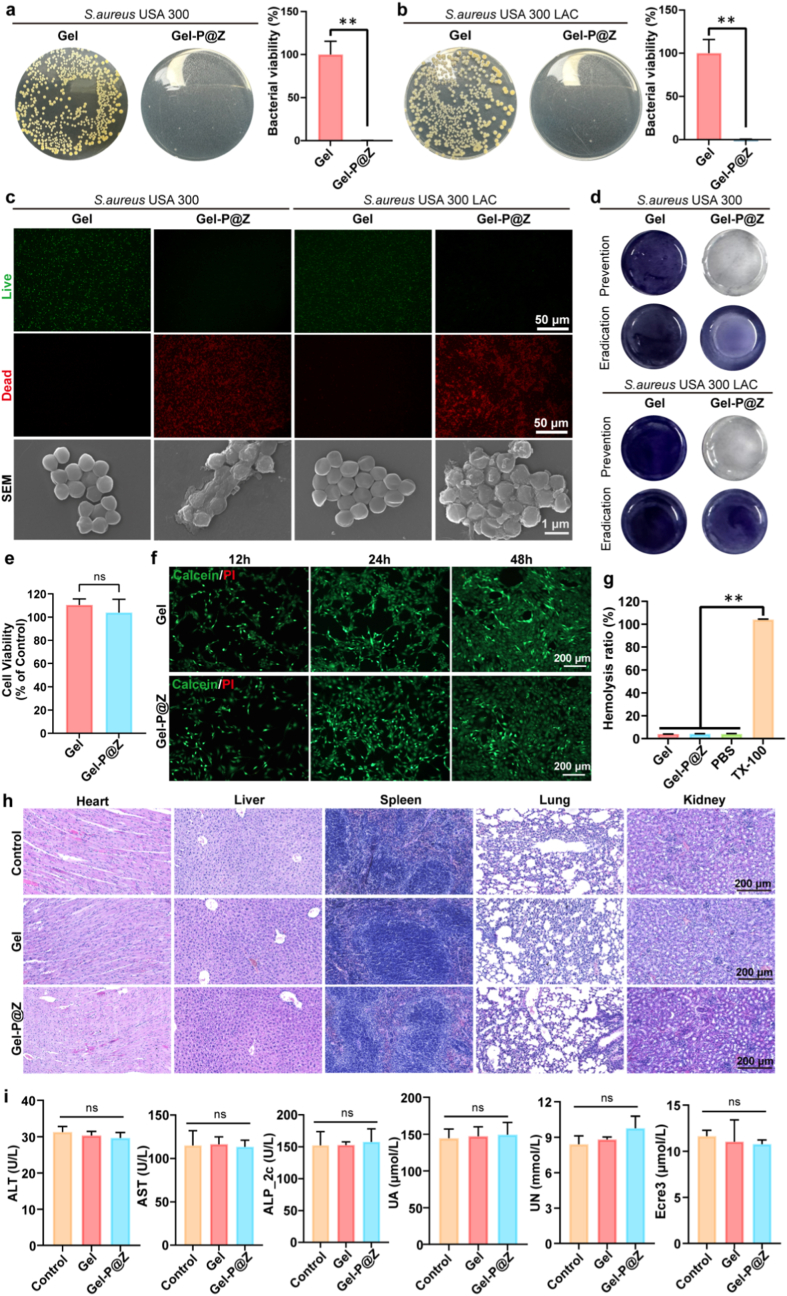
**Antibacterial activity and biocompatibility of Gel-P@Z evaluation *in vitro* and *in vivo***. (a-b) Representative images of bacterial colonies on agar plates after 24 h of co-culture with different hydrogels. Colonies count statistics of MRSA (n = 3). (c) Live/dead fluorescence staining images and SEM images of MRSA in different groups. (d) MTT staining images of MRSA biofilm formation and mature biofilm eradication in different groups. (e) CCK-8 assay of NIH-3T3 cells co-cultured with equal volumes of complete medium and hydrogel-derived extracts after 24 h (n = 4). (f) Live/dead immunofluorescence staining of NIH-3T3 cells. (g) Hemolysis rates under different treatments (n = 3). (h) H&E staining of major organs collected from mice administrated with different treatments. (i) Hepatic and renal function analyses of mice following different treatments (n = 3). Data are presented as mean ± SD. *p < 0.05, **p < 0.01; ns, not significant.

We evaluated the *in vitro* antibacterial activity of Gel-P@Z against two strains of MRSA (*S. aureus* USA 300 and *S. aureus* USA 300 LAC) (Fig. 2a and b). Gel-P@Z hydrogels showed potent antibacterial activity against MRSA. In contrast, Gel control group showed minimal antibacterial activity.

Subsequently, we assessed bacterial viability using a live/dead fluorescence staining assay, where green and red signals correspond to live and dead cells, respectively (Fig. 2c top). As anticipated, the Gel group predominantly exhibited green fluorescence, confirming their minimal effect on bacterial viability. In contrast, the Gel-P@Z group demonstrated a significant eradication of bacteria, indicated by a large red fluorescence area. To further elucidate the damaging effects of hydrogels on bacteria, we employed field emission scanning electron microscopy (FESEM) to examine and analyze the bacterial morphology following cocultivation with hydrogels. The observations revealed that the control group bacteria maintained an intact and smooth surface morphology, suggesting that the viability of the bacteria in this group was preserved (Fig. 2c bottom). Conversely, the bacteria treated with the Gel-P@Z hydrogel displayed ruptured cells and a loss of intact cellular morphology, consistent with our earlier findings regarding the DNA-targeting and reactive oxygen species (ROS)-generating mechanisms of Gly-POX, which disrupt the entire cell [19,26]. This observation visually illustrates the bactericidal mechanism of the Gel-P@Z hydrogel. The Gel-ZIF-8 control group exhibited limited intrinsic antibacterial activity, with residual bacterial survival rates of 48.43% (*S. aureus* USA 300) and 37.84% (*S. aureus* USA 300 LAC), confirming that the core bactericidal performance of the composite hydrogel is mainly attributed to Gly-POX rather than Zn^2+^ from ZIF-8 (Fig. S5).

The ability of Gel-P@Z hydrogel to kill drug-resistant bacteria encouraged us to explore the activity against MRSA biofilms, a formidable threat to human health due to the frequently encountered strong drug resistance. Two essential strategies for combating biofilms involve preventing the formation of biofilms and eradicating mature biofilms. After 24 h of growth, the Gel group displayed a consistent purple film, indicating significant biofilm development (Fig. 2d and Fig. S6). Conversely, the Gel-P@Z groups were almost colorless, suggesting that Gel-P@Z effectively inhibited the formation of biofilm. Simultaneously, the Gel-P@Z group remarkably eliminated mature biofilms. The current study targets chronic wound infections dominated by drug-resistant Gram-positive MRSA, while the performance of this hydrogel against Gram-negative bacterial infections will be systematically explored in future research.

We further investigated the hemocompatibility and cytocompatibility of hydrogels using the extract of hydrogel. The hydrogels showed no obvious cytotoxicity against NIH-3T3 cells (Fig. 2e). Live/Dead staining further confirmed high cell viability across all groups throughout the culture period (Fig. 2f). Moreover, cells maintained normal spindle-shaped morphology and exhibited minimal cell death (red fluorescence) in both hydrogel groups. The supernatant in Gel-P@Z group was cleared and transparent with a hemolysis rate close to 0%, comparable to PBS group, indicating that Gel-P@Z did not result in any apparent hemolysis (Fig. 2g). To evaluate the biosafety of Gel-P@Z for potential clinical translation, hematoxylin and eosin (H&E) staining was performed on major organs (heart, liver, spleen, lung, and kidney). No obvious pathological alterations were observed in the tissues of mice from the experimental groups (Fig. 2h). Additionally, hepatic and renal function parameters in all groups remained within normal physiological ranges (Fig. 2i). In conclusion, the hydrogels have excellent biocompatibility and are safe enough for further *in vivo* experiments.

### 2.3 Gel-P@Z promotes anti-inflammatory M2 macrophage polarization and enhances efferocytosis *in vitro*

**Fig. 3:**
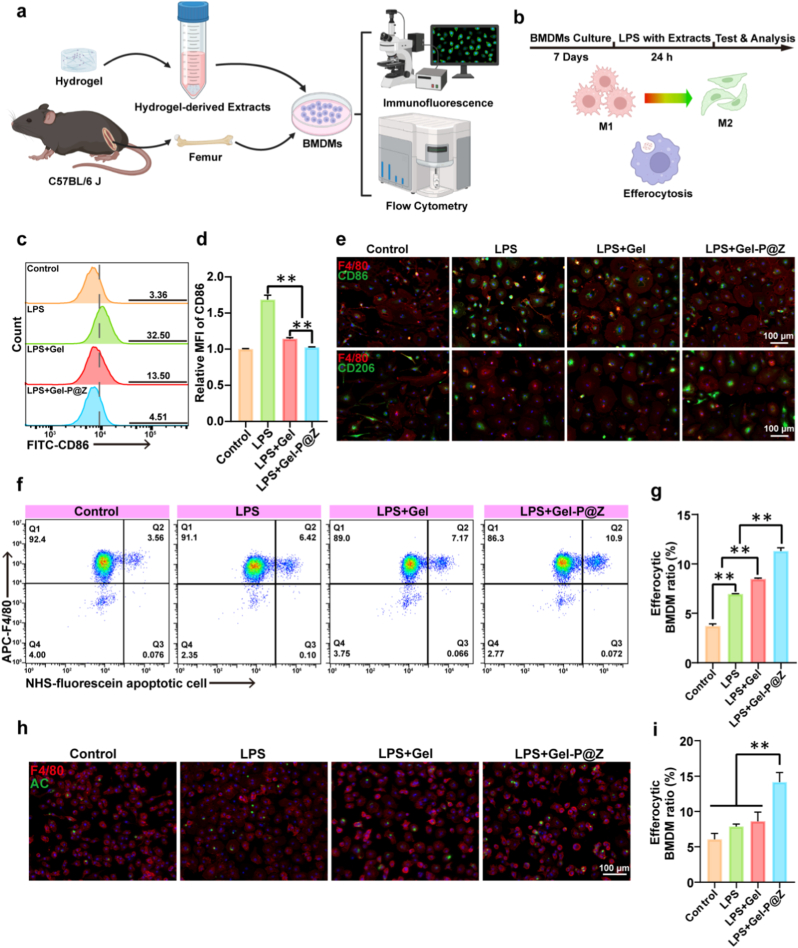
**Gel-P@Z promotes anti-inflammatory M2 macrophage polarization and enhances efferocytosis *in vitro***. (a) Schematic illustration of cell experiments. (b) Workflow diagram of BMDMs polarization and efferocytosis assays. (c) Flow cytometry of CD86 marker on BMDMs. (d) Statistical data of relative MFI of CD86 (n = 6). (e) Immunofluorescence images of CD86, CD206 and F4/80 staining of BMDMs. (f) Flow cytometry of efferocytic BMDMs populations. (g) Statistical data of efferocytic BMDM ratio determined by flow cytometry (n = 5). (h) Immunofluorescence images of efferocytic and non-efferocytic BMDMs. (i) Statistical data of efferocytic BMDM ratio determined by immunofluorescence staining (n = 3). Data are presented as mean ± SD. *p < 0.05, **p < 0.01; ns, not significant.

During the early stages of wound healing, macrophages primarily polarize toward pro-inflammatory M1 or anti-inflammatory M2 phenotypes. The persistent infiltration of M1 macrophages exacerbates inflammation and delays the repair process. Modulating macrophage polarization by suppressing excessive M1 activation and promoting the M2 phenotype may attenuate inflammatory responses and facilitate wound closure [27]. The experiment illustrated in Fig. 3a and b demonstrated the role of Gel-P@Z in driving the phenotypic polarization of macrophages *in vitro*. Specifically, flow cytometry analysis indicated that lipopolysaccharide (LPS) stimulation markedly promoted M1 polarization, as evidenced by a significant increase in the mean fluorescence intensity (MFI) of CD86 compared to the control group (Fig. 3c). In contrast, treatment with Gel-P@Z significantly reduced the MFI of CD86 relative to the LPS group (Fig. 3d), suggesting a shift away from the M1 phenotype. Microscopy images revealed that upon LPS stimulation, most bone marrow-derived macrophages (BMDMs) exhibited a circular shape with dendritic protrusions, whereas a subset of cells adopted an elongated morphology after incubation with Gel-P@Z (Fig. S7). Moreover, immunofluorescence staining showed that compared to the LPS group, the Gel-P@Z group displayed lower fluorescence intensity for CD86 and inducible nitric oxide synthase (iNOS) but higher intensity for CD206 and arginase-1 (Arg-1), collectively indicating a significant shift from M1 to M2 polarization (Fig. 3e and S8). To further corroborate these findings, the mRNA expression of representative M1- and M2-associated cytokines was analyzed by qPCR. As shown in Fig. S9, Gel-P@Z treatment significantly decreased the expression of the pro-inflammatory genes *Tnf* and *Il6*, whereas the anti-inflammatory genes *Il10* and *Il4* were markedly upregulated compared with the LPS group. These transcriptional changes further support the ability of Gel-P@Z to attenuate inflammatory activation and promote macrophage polarization toward the M2 phenotype. This polarization effect may be partially attributed to Zn^2+^ ions released from ZIF-8 degradation, which are known to attenuate macrophage-driven inflammatory responses and promote differentiation toward the anti-inflammatory M2 phenotype [28].

Given that infected wounds contain abundant apoptotic cells and cellular debris, we evaluated the efferocytic capacity of macrophages following incubation with Gel-P@Z. Etoposide-treated (12 h) HL60 cells were utilized as apoptotic cells (AC), labeled with NHS-fluorescein, and subsequently added to BMDMs from different treatment groups (Fig. 3a and b). Flow cytometry analysis revealed that the efferocytic ratio of BMDMs in the LPS + Gel-P@Z group was approximately 10.9%, which was significantly higher than that in the LPS-only group (∼6.42%) (Fig. 3f), representing a statistically significant increase of approximately 4.48% (Fig. 3g). Immunofluorescence staining further confirmed that BMDMs incubated with Gel-P@Z under the LPS stimulation engulfed a greater number of NHS-fluorescein-labeled apoptotic cells (Fig. 3h and i). Collectively, these results indicate that Gel-P@Z enhances the efferocytic capacity of macrophages, thereby facilitating the effective clearance of apoptotic cells within infected wounds. Previous studies have reported that Zn^2+^ ions can upregulate efferocytosis-related proteins (TSP1, CD36, MERTK, Arg-1, and ODC-1), thereby effectively enhancing efferocytosis [29]. Additionally, it has been reported that macrophages undergo phenotypic remodeling toward the anti-inflammatory M2 repair phenotype after completing efferocytosis, accompanied by the secretion of various pro-repair factors, including TGF-β1 and angiogenin, which consequently promote fibroblast activation and angiogenesis [30]. Therefore, the enhanced efferocytosis observed with Gel-P@Z in this study may also be attributed to the Zn^2+^ released from ZIF-8 degradation. Furthermore, the subsequent transition to the M2 phenotype following efferocytosis corroborates the finding that Gel-P@Z promotes macrophage polarization from the M1 to the M2 phenotype.

### 2.4 Gel-P@Z promotes fibroblast cell migration *in vitro* and fascia explants contraction *ex vivo*

**Fig. 4:**
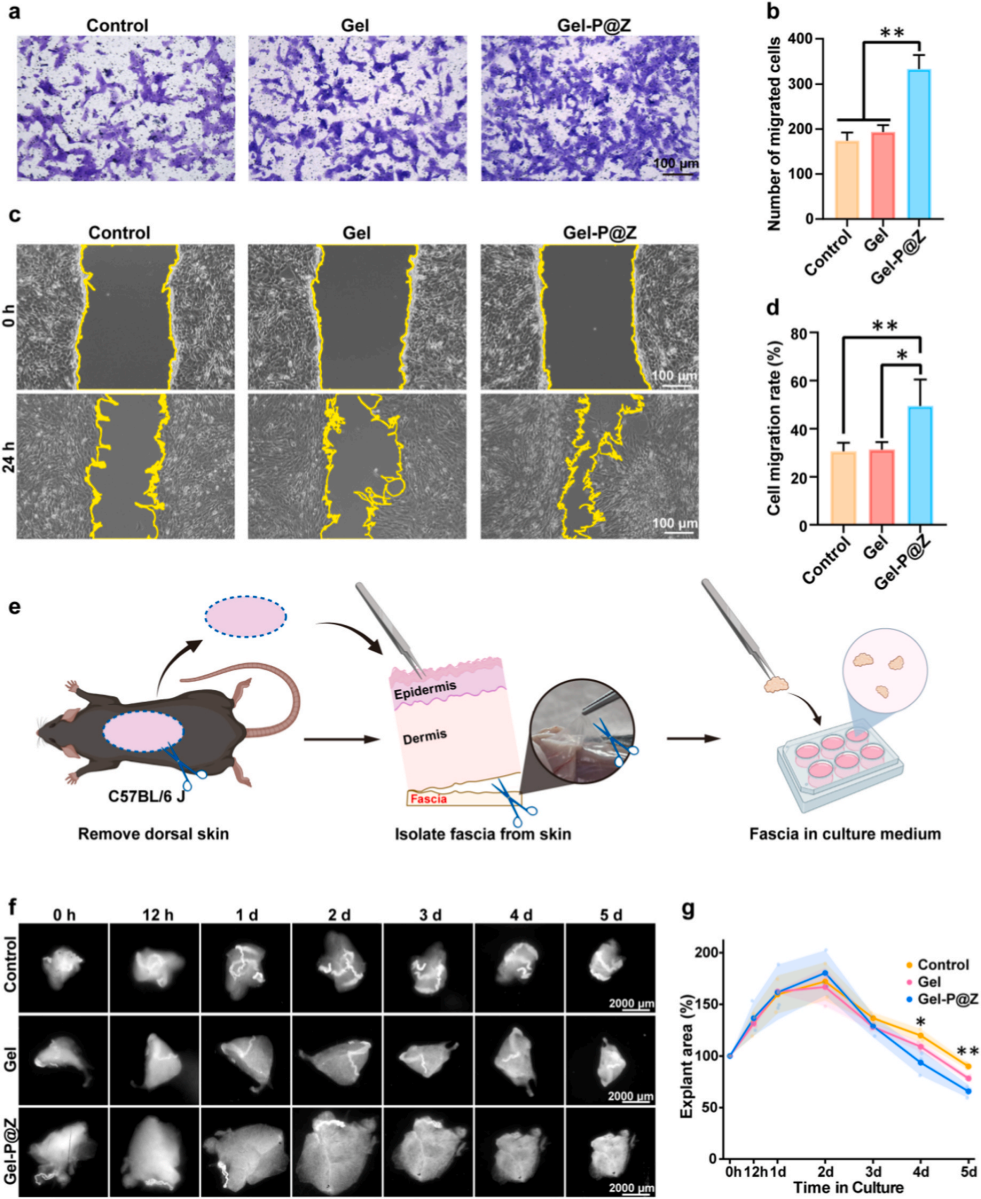
**Gel-P@Z promotes fibroblast cell migration *in vitro* and fascia explant contraction *ex vivo***. (a) Transwell assays of NIH-3T3 cells at 24 h. (b) Statistical data of number of migrated cells (n = 3). (c) Scratch assays of NIH-3T3 cells at 0 and 24 h. (d) Statistical data of cell migration rate (n = 3). (e) Schematic illustration of isolating fascia from skin. (f) Bright-field images of fascia explants under different treatments. (g) Fascia explant area over time (n = 3). Data are presented as mean ± SD. *p < 0.05, **p < 0.01; ns, not significant.

Fibroblast recruitment and migration play a vital role in wound healing. To evaluate the potential of the hydrogels in promoting cell migration, we performed both transwell and scratch wound healing assays using NIH-3T3 fibroblasts. The transwell migration assays demonstrated that treatment with Gel-P@Z significantly enhanced cell migration, resulting in a nearly 2-fold increase in the number of migrated cells compared to both the control and the pure Gel groups (Fig. 4a and b). Scratch wound assays further corroborated this trend (Fig. 4c and d). To further assess the effects of Gel-P@Z on fibroblast activation and extracellular matrix remodeling, the mRNA expression of representative ECM remodeling-related genes was examined by qPCR. As shown in Fig. S10, Gel-P@Z significantly increased the expression of *Col1a1* and *Acta2* compared with both the control and Gel groups. The upregulation of these genes, which are associated with collagen synthesis and myofibroblast differentiation, suggests that Gel-P@Z facilitates extracellular matrix remodeling in addition to promoting fibroblast migration. Collectively, these results indicate that Gel-P@Z promotes fibroblast recruitment and migration, which may contribute to accelerated wound healing. This pro-migratory effect of Gel-P@Z may be attributed to the release of Zn^2+^ ions resulting from ZIF-8 degradation, as previous studies have shown that Zn^2+^ functions as a known activator of fibroblast motility [[31], [32], [33]].

Fascia mobilization plays a crucial role in wound healing, with its subsequent contraction accelerating wound closure in the later phases [34]. Specifically, fascia fibroblasts organize into sprout-reticulate-cluster assemblies, coordinating migration and division [35]. Upon injury, these fibroblasts undergo swarming-like collective migration, pulling the surrounding extracellular jelly-like matrix along with embedded blood vessels, macrophages, and peripheral nerves to form a provisional matrix that progressively contracts and promotes healing [36]. We adopted a skin-fascia explant model and quantified contraction by measuring the change in explant area over time as outlined in the experimental scheme [37] (Fig. 4e). Bright-field images revealed that fascia explants in all treatment groups exhibited an initial swelling followed by contraction at the indicated time points (Fig. 4f). In contrast, fascia explants in the Gel-P@Z group exhibited the most pronounced effects on both swelling and contraction compared to the other groups (Fig. 4g). The observed enhancement in fascial contraction may be attributed to Zn^2+^ ions released during ZIF-8 degradation, suggesting a mechanism whereby Zn^2+^ ions could facilitate the transition of fascia fibroblasts toward contractile myofibroblasts, thereby promoting tissue contraction and wound closure [37]. In conclusion, these results indicate that Gel-P@Z supports wound healing by enhancing the recruitment and migration of fascia fibroblasts.

### 2.5 Gel-P@Z hydrogel facilitates rapid healing of MRSA-infected wounds in mice

**Fig. 5:**
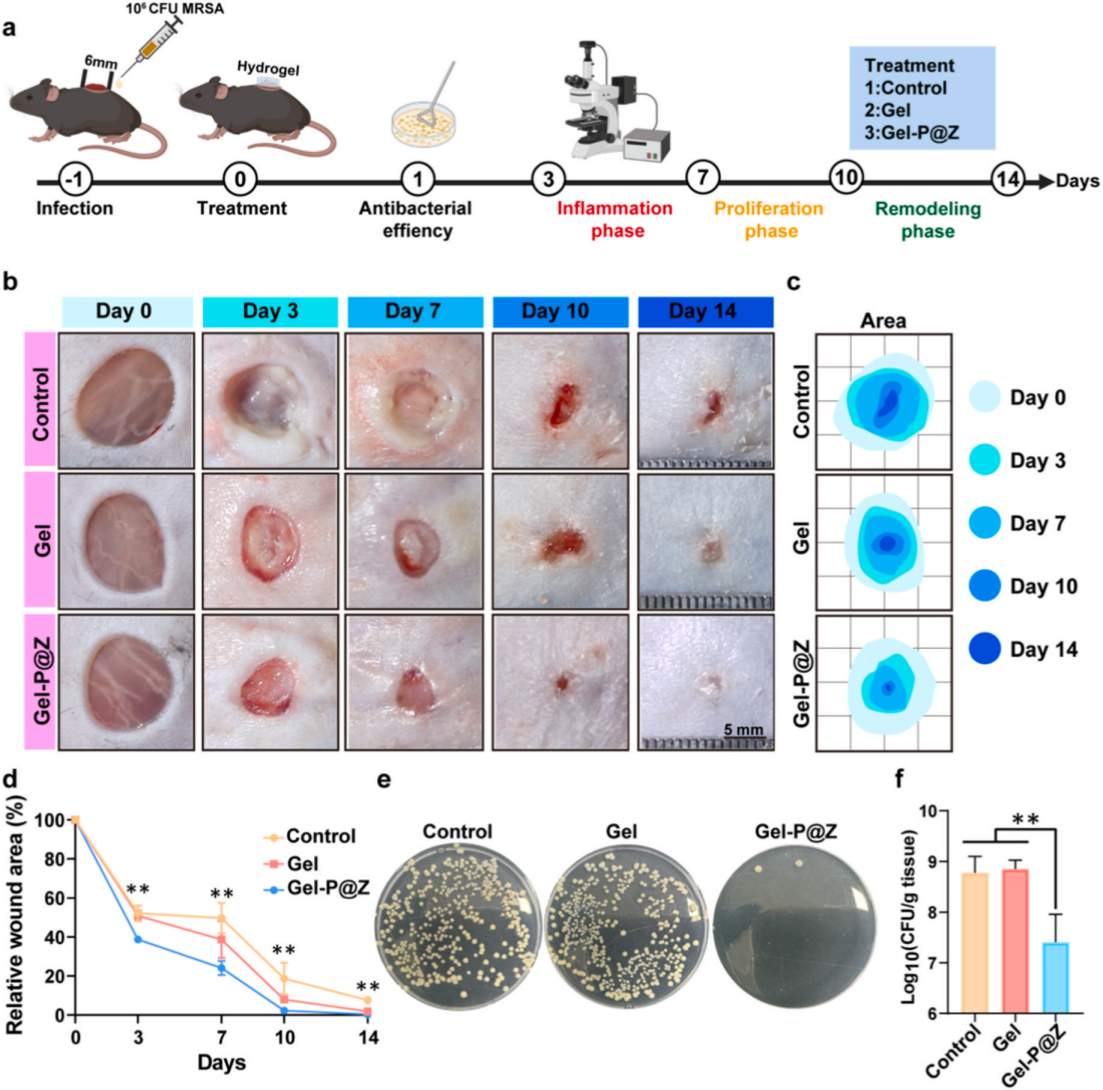
**Gel-P@Z hydrogel facilitates rapid healing of MRSA-infected wounds in mice**. (a) Schematic illustration of the treatment protocol for the MRSA-infected wound model in mice. (b) Representative photographs of wound closure changes from day 0 to day 14. (c) Simulation plots of wound closure. (d) Variation curve of wound areas within 14 days (n = 4). (e) Representative photographs of bacterial colonies cultured from wounds on day 1. (f) Statistical data of bacterial colony count (n = 3). Data are presented as mean ± SD. *p < 0.05, **p < 0.01; ns, not significant.

Encouraged by the potent antibacterial activity and diverse cellular functions *in vitro* of nanocomposite hydrogels, we proceeded to evaluate the *in vivo* wound healing efficacy using a MRSA-infected mouse full-thickness wound model (Fig. 5a). Two full-thickness excisional wounds (round, 6.0 mm) were created on the back of C57BL/6J mice and infected with MRSA, followed by treatments with saline (Control), Gel and Gel-P@Z, respectively. Digital photographs clearly indicated a markedly accelerated wound closure rate in the Gel-P@Z group (Fig. 5b and c). Quantification of the wound areas further confirmed that the Gel-P@Z group exhibited the fastest closure kinetics and the smallest residual wound area at all observed time points, whereas control wounds exhibited delayed healing, as expected for MRSA-infected wounds (Fig. 5d).

In parallel, we assessed the *in vivo* antibacterial activity of the hydrogels. After 1 day treatment with hydrogels, the bacteria in the control and Gel groups continued to proliferate, while the Gel-P@Z treatment most effectively reduced bacterial counts (Fig. 5e). The Gel-P@Z group exhibited ∼1.44 log reduction of CFU at the wound lesion, which is better than control and Gel hydrogels (Fig. 5f). This finding is consistent with the *in vitro* antibacterial test results, indicating that Zn^2+^ ions from ZIF-8 and Gly-POX synergistically contribute to the antibacterial efficacy of the hydrogel.

**Fig. 6:**
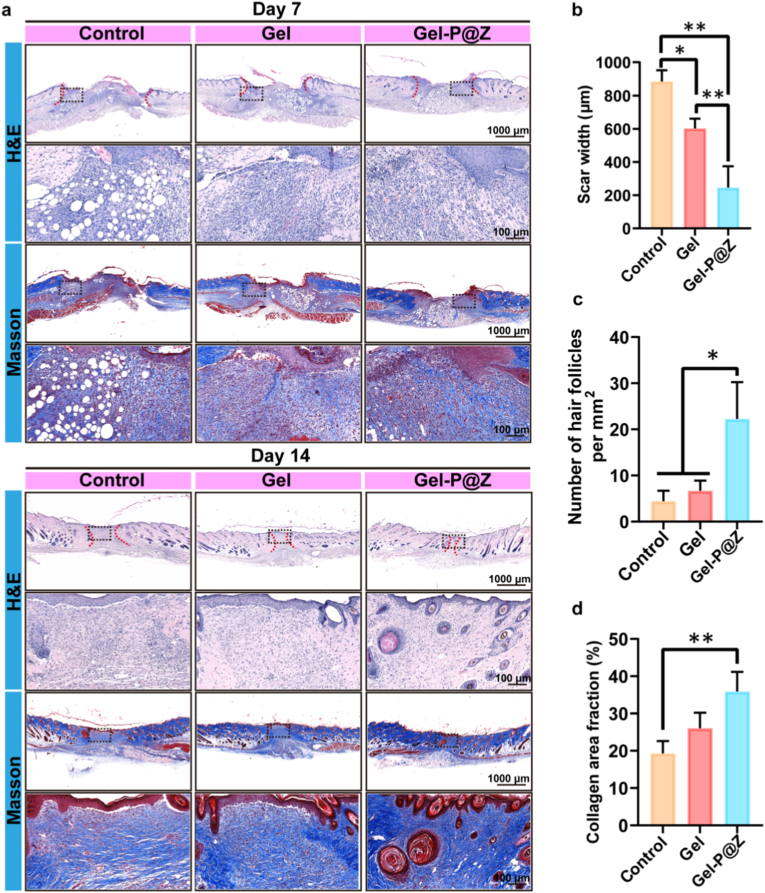
**Gel-P@Z hydrogel facilitates rapid healing of MRSA-infected wounds in mice**. (a) H&E staining and Masson's trichrome staining images of wound tissues at day 7 and 14. (b) Statistical analysis of scar width on day 14 based on H&E staining (n = 3). (c) Quantification of hair follicle density on day 14 based on H&E staining (n = 3). (d) Statistical analysis of collagen area fraction on day 14 based on Masson's trichrome staining (n = 3). Data are presented as mean ± SD. *p < 0.05, **p < 0.01; ns, not significant.

During the wound healing phase, the structural characteristics of skin tissue were evaluated using H&E staining and Masson's trichrome staining (Fig. 6a). H&E staining on day 7 showed pronounced inflammatory cell infiltration in the control and Gel groups, while fewer inflammatory cells were observed in the Gel-P@Z group. Notably, inflammatory cell infiltration persisted in the control group until day 14. Both H&E and Masson's trichrome staining confirmed complete re-epithelialization in all groups by the end of the observation period. Furthermore, compared with the Control and Gel groups, the Gel-P@Z group exhibited a significantly smaller scar width on day 14 (Fig. 6b), along with the presence of a limited number of newly formed hair follicles (Fig. 6c). Masson's trichrome staining further demonstrated that collagen deposition in the Gel-P@Z group was more mature and well-organized relative to the other treatment groups (Fig. 6d). Collectively, these findings suggested that Gel-P@Z effectively inhibits bacteria in *MRSA*-infected wounds and promotes wound healing.

### 2.6 Gel-P@Z promotes wound healing via macrophage polarization, efferocytosis, cellular proliferation, angiogenesis and collagen deposition

**Fig. 7:**
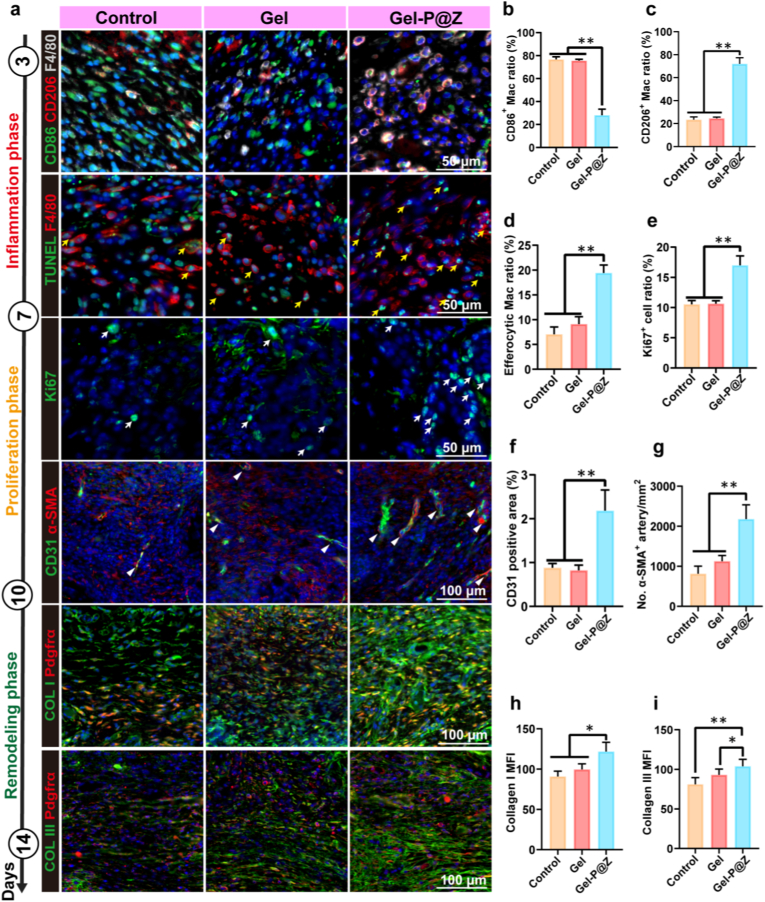
**Gel-P@Z promotes wound healing via macrophage polarization, efferocytosis, cellular proliferation, angiogenesis and collagen deposition**. (a) Representative immunofluorescent staining of CD86 (green), CD206 (red), F4/80 (white/red), TUNEL (green), Ki67 (green), CD31 (green), α-SMA (red), COL I (green), COL III (green), PDGFRA (red) and DAPI (blue) images of wound tissue in different wound healing phases. (b-c) Quantification of CD86^+^ and CD206^+^ macrophage ratios on day 3 (n = 4). (d) Efferocytic macrophage ratio on day 3, determined by co-localization of F4/80 and TUNEL signals (n = 4). (e) Statistical data of Ki67^+^ cell ratio on day 7 (n = 4). (f-g) Statistical data of CD31 positive area ratio and α-SMA positive area on day 10 (n = 4). (h-i) Statistical data of mean fluorescent intensity of COL I and COL III on day 14 (n = 3). Data are presented as mean ± SD. *p < 0.05, **p < 0.01; ns, not significant. (For interpretation of the references to color in this figure legend, the reader is referred to the Web version of this article.)

To systematically evaluate healing quality, tissue samples were collected on day 3, 7, 10 and 14 for immunofluorescence analysis. Macrophages play a critical role in the inflammatory stage and are broadly categorized into pro-inflammatory M1 and anti-inflammatory M2 phenotypes within the wound environment. Immunofluorescence staining showed fewer M1-type (CD86^+^) and more M2-type (CD206^+^) macrophages at the wound sites in the Gel-P@Z group compared with the Control and Gel groups at day 3, suggesting the reduction of inflammatory response and the repolarization of macrophages from the M1 to the M2 phenotype (Fig. 7a, b and 7c). Additionally, MRSA-infected skin wounds are characterized by a substantial presence of apoptotic cells during the inflammation phase, leading to intense inflammation and oxidative stress [38,39]. Therefore, macrophage-mediated efferocytosis of these cells is essential for mitigating damage [40]. We performed IF co-staining of the macrophage marker F4/80 and the apoptosis marker TUNEL. A higher proportion of F4/80^+^TUNEL^+^ macrophages was observed in the Gel-P@Z group compared to the control and Gel groups, indicating that Gel-P@Z enhanced the efferocytic capacity of macrophages. (Fig. 7a and d).

The proliferative phase is a critical stage in wound healing, including cellular proliferation and neovascularization. On day 7, Gel-P@Z hydrogels induced a greater number of Ki67^+^ proliferating cells compared to the other groups, indicating an enhanced generation of newly generated cells to support re-epithelialization and granulation tissue formation (Fig. 7a and e). Neovascularization was comprehensively evaluated in wound tissues on day 10 via immunofluorescence staining for CD31 (a marker of early angiogenic endothelial cells) and α-SMA (a marker indicating perivascular cell recruitment and reflecting vascular maturation). The CD31^+^ area in the Gel-P@Z group was significantly larger than that in the other groups (Fig. 7a and f). Furthermore, immunofluorescence co-staining of α-SMA and CD31 confirmed a greater presence of arterioles in the Gel-P@Z group, consistent with its enhanced neovascularization (Fig. 7a and g).

Collagen deposition is a key process during the remodeling phase of wound healing. Type I collagen primarily provides structural integrity, while type III collagen contributes to tissue elasticity [41]. The expression levels of both collagen I and collagen III were significantly increased in the Gel-P@Z group, consistent with the results of Masson's trichrome staining, which confirmed the successful progression of wound repair and regeneration (Fig. 7a, h and 7i). The recruitment and migration of fibroblasts to the wound site underlie collagen deposition in the wound bed. Correspondingly, immunofluorescence analysis revealed a higher number of PDGFRα^+^ fibroblasts in the Gel-P@Z group compared to the Control and Gel groups. This observation further supports the findings from the transwell and scratch assays. It is worth noting that the Gly-POX peptide mimic primarily exerts bactericidal effects, with no intrinsic anti-inflammatory activity or regulatory function in fibroblast migration. Further systematic mechanistic research will be conducted to clarify the specific contributions of each component in subsequent studies.

Collectively, these coordinated actions significantly accelerate the repair and regeneration of MRSA-infected wounds, demonstrating considerable clinical promise.

### 2.7 Gel-P@Z improves wound healing via TGF-β signaling pathway

**Fig. 8:**
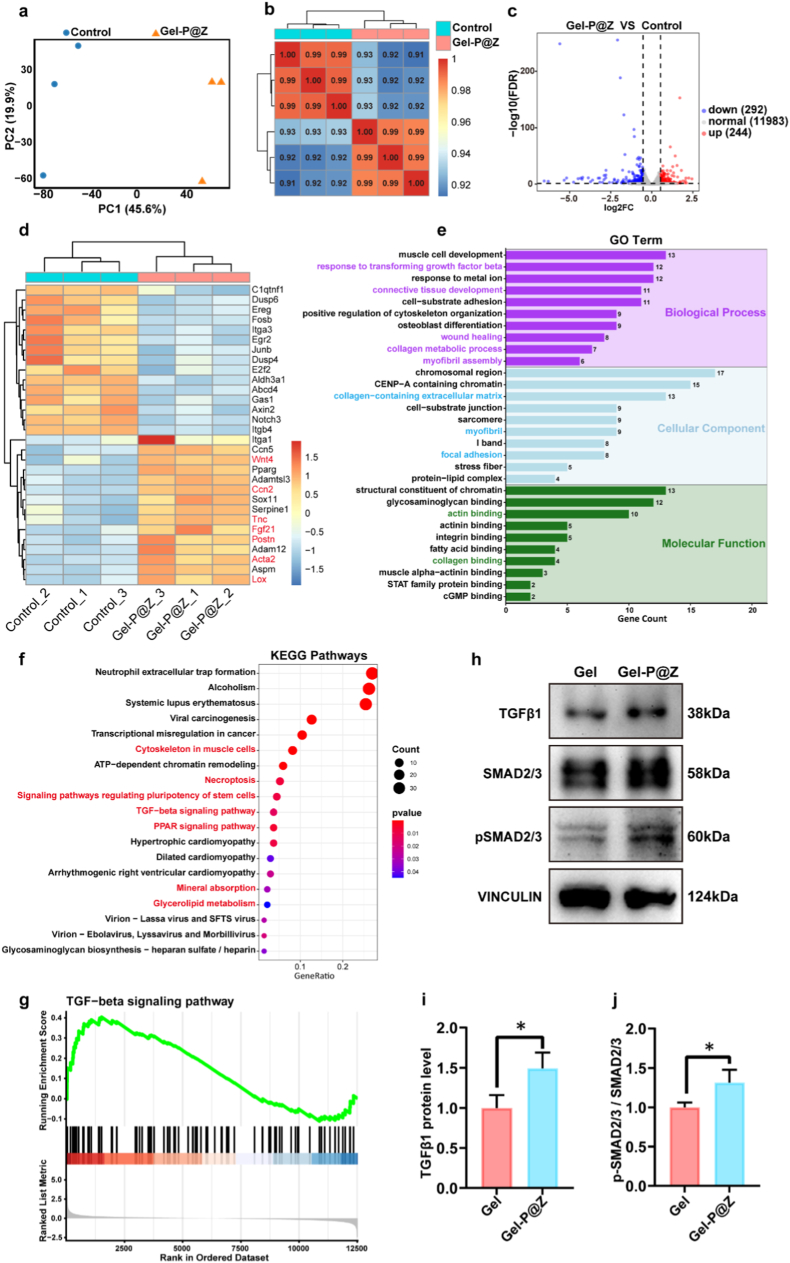
**Gel-P@Z improves wound healing via TGF-β signaling pathway**. (a) Principal component analysis (PCA) of the transcriptomic data. (b) Heatmap of Pearson's correlation coefficient matrix for tested samples. (c) Volcano plots illustrating differentially expressed genes (DEGs) between Gel-P@Z and control groups. (d) Heatmap of the DEGs. (e) Gene Ontology (GO) enrichment analysis of DEGs. (f) Kyoto Encyclopedia of Genes and Genomes (KEGG) analysis of DEGs. (g) Gene Set Enrichment Analysis (GSEA) plot of the TGF-β signaling pathway. (h) Representative Western Blot images showing the expression of TGFβ1, SMAD2/3, p-SMAD2/3 and VINCULIN in NIH-3T3 under different treatments. (i) Quantification of TGFβ1 protein expression normalized to VINCULIN (n = 3). (j) Phosphorylation level of Smad2/3 (n = 3). Data are presented as mean ± SD. *p < 0.05, **p < 0.01; ns, not significant.

To further explore the mechanism by which Gel-P@Z hydrogel promotes wound healing, bulk RNA sequencing (RNA-seq) was performed to analyze its effects on gene expression in NIH-3T3 fibroblasts. Principal component analysis (PCA) showed high reproducibility and strong correlation across samples (Fig. 8a). All biological replicates exhibited a squared Pearson correlation coefficient (R^2^) above 0.91, confirming sample reliability (Fig. 8b). Volcano plot analysis identified 536 differentially expressed genes (DEGs) in the Gel-P@Z group relative to the control, with 244 up-regulated and 292 down-regulated (Fig. 8c). Gene clustering heatmaps revealed that Gel-P@Z up-regulated several genes including *Wnt4*, *Ccn2*, *Tnc*, *Fgf21*, *Postn*, *Acta2*, and *Lox*, functionally associated with extracellular matrix formation, cell migration, and cell adhesion (Fig. 8d). Gene Ontology (GO) analysis indicated that the DEGs were primarily enriched in biological processes related to cellular response to TGF-β stimulation, connective tissue development, collagen metabolism, and myofibril assembly (Fig. 8e). Correspondingly, Kyoto Encyclopedia of Genes and Genomes (KEGG) pathway enrichment analysis revealed significant enrichment of the TGF-β signaling pathway and PPAR signaling pathway among the differentially expressed genes (Fig. 8f). Notably, the TGF-β signaling pathway was consistently identified in both GO and KEGG analyses, underscoring its central role in the transcriptional response to Gel-P@Z treatment. To validate this finding, Gene Set Enrichment Analysis (GSEA) was conducted focusing on the TGF-β signaling pathway (mmu04350). The results demonstrated significant up-regulation of the TGF-β signaling pathway in the Gel-P@Z group relative to the control group (Fig. 8g). To further validate the transcriptomic findings at the protein level, Western blot analysis was performed to examine the activation of the TGF-β signaling pathway. As shown in Fig. 8h–j, Gel-P@Z treatment significantly increased the protein level of TGFβ1 compared with the Gel group. Notably, Gel-P@Z significantly enhanced SMAD2/3 phosphorylation, resulting in an increased p-SMAD2/3/SMAD2/3 ratio. Since phosphorylation of SMAD2/3 is a hallmark of canonical TGF-β signaling activation, these findings provide direct evidence that Gel-P@Z activates the TGF-β/SMAD pathway in fibroblasts, thereby corroborating the transcriptomic analyses. Previous studies have demonstrated that TGF-β is a key regulator of fibrosis, and the TGF-β signaling pathway plays a critical role in wound healing and fibrotic processes [42]. The underlying mechanism primarily involves TGF-β binding to TGFβR2, recruiting and phosphorylating TGFβR1, which then phosphorylates SMAD2/3. The activated SMAD2/3 forms a complex with SMAD4 and translocates into the nucleus, where it regulates the expression of genes such as collagen, α-SMA and TIMP. This cascade ultimately promotes the transformation of fibroblasts into myofibroblasts, leading to the accumulation of extracellular matrix (ECM) [43]. In summary, these transcriptomic findings and protein-level analyses demonstrate that Gel-P@Z activates the canonical TGF-β/SMAD signaling pathway in fibroblasts, thereby promoting fibrosis-related gene expression, myofibroblast differentiation, extracellular matrix deposition, and ultimately accelerating wound healing.

## 3 Conclusion

In summary, we successfully loaded the antibacterial-mimetic polymer Gly-POX onto the ZIF-8 metal-organic framework and incorporated the resulting composite into a gelatin hydrogel to form the multifunctional Gel-P@Z system. The degradation of the gelatin matrix and ZIF-8 enabled controlled release of Gly-POX, which achieved potent antibacterial activity against MRSA (>99%). Both *in vitro* and *in vivo* studies confirmed the excellent biocompatibility of Gel-P@Z, supporting its suitability for biomedical applications. The released Zn^2+^ ions promoted macrophage polarization from the pro-inflammatory M1 to the anti-inflammatory M2 phenotype, thereby shaping an immunomodulatory microenvironment and enhancing efferocytic capacity. Additionally, Gel-P@Z enhanced fibroblast migration by approximately 2-fold and upregulated extracellular matrix-related gene expression. Interestingly, Gel-P@Z treatment induced a rapid swelling followed by contraction of the fascia explants, suggesting its ability to recruit fascia fibroblasts and enhance fascial contraction, highlighting its considerable potential in wound healing. In a murine model of MRSA-infected wounds, Gel-P@Z accelerated healing and promoted macrophage polarization, efferocytosis, cellular proliferation, angiogenesis and collagen deposition. Integrated transcriptomic and protein-level analyses further revealed that Gel-P@Z enhanced wound healing through activation of the TGF-β signaling pathway. Taken together, these findings underscore the multifunctional efficacy of Gel-P@Z in infected wounds, showing great potential for advanced wound care applications.

## 4 Method

### 4.1 Synthesis and characterization of peptide-mimicking poly(2-oxazoline) Gly-POX

In a nitrogen-filled glovebox, an oven-dried reaction vial equipped with a magnetic stir bar was charged with *N*-Boc-aminomethyl-2-oxazoline (100 mg) and *N,N*-dimethylacetamide (DMAc, 500 μL). The mixture was stirred until a homogeneous solution was obtained. Methyl trifluoromethanesulfonate solution (MeOTf, 1 M, 25 μL) was then added as the initiator, the vial was immediately sealed, and the reaction mixture was heated at 120°C with stirring overnight. The progress of the polymerization was monitored by thin-layer chromatography (TLC). After complete consumption of the monomer, the reaction mixture was diluted with tetrahydrofuran (THF, 0.5 mL), followed by the addition of *n*-hexane to induce precipitation of the polymer. The suspension was centrifuged and the supernatant was decanted; this dissolution–precipitation–centrifugation procedure was repeated three times. The resulting precipitate was treated with trifluoroacetic acid (TFA, 2 mL) and shaken at room temperature for 2 h. Thereafter, most of the TFA was removed under a gentle stream of nitrogen, and the residue was dissolved in methanol (MeOH, 0.5 mL). Methyl tert-butyl ether was added to induce precipitation, and the suspension was centrifuged and the supernatant discarded. The obtained solid was dried under vacuum in a desiccator, redissolved in ultrapure water (5 mL), filtered, and finally lyophilized. Poly(2-oxazoline) at the NHBoc protected stage was characterized by GPC using *N,N*-dimethyl formamide (DMF), supplemented with 0.01 M LiBr, as the mobile phase at a flow rate of 1 mL/min at 50°C. Sample solutions were filtered through 0.22 μm polytetrafluoroethylene filters before GPC analysis. The relative number average molecular weight (Mn) and dispersity index (*Đ*) were calculated from a calibration curve using poly(methylmethacrylate) PMMA as internal standards on a Breeze 2 software. Degree of polymerization (DP) was determined from the obtained *Mn* in GPC analysis. The final Gly-POX was confirmed by proton nuclear magnetic resonance (^1^H NMR) characterization.

### 4.2 The preparation and characterization of ZIF-8 and Gly-POX@ZIF-8

To synthesize ZIF-8 nanoparticles (NPs), Zn(NO_3_)_2_·6H_2_O (15 mmol) and 2-MeIm (60 mmol) were dissolved in 150 mL of methanol to prepare the precursor solution, respectively. The 2-MeIm solution was then added dropwise into the zinc nitrate solution under stirring. The reaction mixture was stirred for 15 min at rt at a speed of 800 rpm. Finally, the product was washed with methanol and deionized water through repeated centrifugation and redispersion, followed by centrifugation at 10,000 rpm for 10 min, and vacuum-drying. To prepare the Gly-POX-loaded ZIF-8 NPs (Gly-POX@ZIF-8), a suspension was created by dispersing an equal mass ratio of the synthesized ZIF-8 NPs and Gly-POX powder in 1 mL of PBS. This mixture was then vortexed and subjected to ultrasonication for 30 min. Finally, the functional ZIF-8 powder was collected through freeze-drying. The morphology of the nanoparticles was characterized using transmission electron microscope (TEM) operating at 200 kV. Size distribution and zeta potential were measured with a dynamic light scattering (DLS) analyzer. The characterization of Gly-POX@ZIF-8 was further performed using Fourier-Transform infrared spectroscopy spectra, TGA and X-ray diffraction analysis.

### 4.3 The preparation and characterization of Gel and Gel-P@Z hydrogels

**Preparation.** To prepare the nanocomposite GelMA hydrogel scaffolds, nanoparticles at a concentration of 2 mg/mL were initially dispersed in PBS using ultrasonic dispersion. GelMA nanocomposite hydrogels were prepared by mixing 15% (w/v) GelMA with the nanoparticles Gly-POX@ZIF-8 followed by photo-crosslinking, and were hereafter denoted as Gel-P@Z. Likewise, pure GelMA hydrogels, which were photocrosslinked under identical conditions, were denoted Gel. GelMA was dissolved in PBS containing 0.5% (w/v) of the photoinitiator lithium phenyl-2,4,6-trimethylbenzoylphosphinate (LAP) and allowed to stand for 30 min to obtain a colorless liquid at 35°C. Subsequently, the nanoparticle dispersions were added to the GelMA pre-solution. 50 μL of the nanocomposite GelMA precursor solution was added to an 8 well-plate coverslip glass and further exposed to ultraviolet light for 1 min.

***In vitro* release behavior.** The release of Gly-POX from the 3D Gel scaffold was analyzed by fluorescamine protein assay. In brief, Gel-P@Z hydrogels were immersed in either PBS (pH = 7.4) or PBS (pH = 6.0). At predetermined time points, the samples were collected and incubated with fluorescamine at concentration of 1 mM for 15 min before reading fluorescence intensity on the plate reader.

**Swelling ratio.** Swelling ratio of the freeze-dried hydrogel was measured gravimetrically. In brief, a certain mass (W_0_) of freeze-dried hydrogels was immersed in PBS (pH = 7.4) or PBS (pH = 6.0). At predetermined time points, the swollen hydrogels were removed and weighed (Wt) after the excess of PBS lying on the surface was absorbed with a filter paper. The equilibrium of swelling was achieved when the weights of samples kept constant. All experiments were repeated three times. The swelling ratio (%) was calculated using the following equation:

$$\text{SR}\left(\%\right)=\left(W_{t}-W_{0}\right)/W_{0}\times 100\%$$

**Morphology.** The morphology of the Gel hydrogel was characterized using scanning electron microscopy (SEM). The hydrogel was subjected to gelation followed by freeze-drying. After sputter-coating with a conductive layer, the morphologies were viewed using an S4800 SEM operated at 5.0 kV accelerating voltage.

***In vitro* degradation behavior.** The *in vitro* degradation of Gel and Gel-P@Z hydrogels was examined gravimetrically under simulated physiological conditions. Specifically, 50 μL of the hydrogel was lyophilized and weighed (W_0_). Weight loss was monitored as a function of incubation time in PBS (pH = 7.4) or PBS (pH = 6.0) at rt. At specific time points, the samples were carefully withdrawn from the PBS, freeze-dried and subsequently weighed (Wt). The weight loss percentage (ΔW %) was defined as follows:

$$\Delta W\left(\%\right)=W_{t}/W_{0}\times 100\%$$

### 4.4 Antibacterial assay

Methicillin-resistant *Staphylococcus aureus* (*S. aureus* USA300 and *S. aureus* USA300 LAC) were utilized to evaluate the antibacterial activity of the hydrogel, following the previous protocol [44,45]. Briefly, the bacterial suspension was adjusted to a cell density of 10^6^ CFU/mL, serving as the working solution for the antimicrobial assay. Individual hydrogels were placed in a 48-well plate and evenly covered with 100 μL of the bacterial working solution. After incubation at 37°C for 3 h, an aliquot of 900 μL of PBS was added to each well of the 48-well plate. The plate was then sonicated for 3 min and vortexed for 2 min to detach bacteria from the hydrogels. To determine the colony count C_sample_, 20 μL of the bacterial suspension from each well was spread onto agar plates, followed by overnight incubation at 37°C. A bacterial suspension that had not been incubated on the hydrogel served as the negative control, providing the colony count C_control_. The bacterial viability of the hydrogels was calculated using the following equation:

$$\text{Bacterial}\;\text{viability}\;\left(\%\right)=C_{\text{sample}}/C_{\text{control}}\times 100$$

**Live/dead staining.** In order to evaluate the bactericidal effect, a live/dead staining procedure was conducted. Propidium iodide (PI) was used to label dead bacteria, whereas SYTO 9 stained live bacteria, resulting in red and green fluorescence, respectively. In summary, after incubating with hydrogels for 3 h, the medium was centrifuged and replaced with fresh PBS containing 10 μg/mL PI and 1.25 μM SYTO 9. Following a 15 min incubation at 37°C, the bacterial fluorescence was observed under a fluorescence microscope.

**SEM characterization of bacteria morphology.** The bacterial suspension was adjusted to a cell density of 1 × 10^7^ CFU/mL. Individual hydrogels were placed in 1.5 mL tubes and evenly covered with 1 mL of the bacterial solution. After co-culturing at 37°C for 3 h, the bacteria were collected by centrifugation at 4000 rpm for 5 min, then resuspended in 1 mL of PBS buffer and centrifuged again to collect the bacteria. The bacteria were fixed overnight at 4°C after the addition of 1 mL of PBS buffer containing 2.5% glutaraldehyde, followed by thorough vortexing. The next day, the bacteria were collected by centrifugation and washed three times with PBS, followed by gradient dehydration using ethanol at various concentrations (30%, 50%, 70%, 80%, 90%, 95%, and 100%). The dehydrated bacteria were dispersed in 20 μL of absolute ethanol, and the resulting ethanol solution containing the bacteria was then dropped onto the surface of a silicon wafer. After natural air drying, SEM observation was conducted.

**Inhibition study on biofilm formation.** *S. aureus* USA300 and *S. aureus* USA300 LAC were cultured in Luria-Bertani (LB) medium at 37°C for 12 h, and then the bacterial suspension was diluted with Mueller Hinton (MH) (containing 1% glucose) medium to a cell density of 2 × 10^6^ CFU/mL as the working suspension. MH medium (50 μL) containing hydrogel extracts was performed in a tissue culture treated 96-well plate. 50 μL of bacterial suspension was added to each well, followed by moderate shaking of the plate for several seconds. The plate was kept at 37°C for 24 h. After removing the culture solution, 100 μL of MTT solution (0.5 mg/mL) was added into each well and the plate was incubated for 4 h in the dark. The supernatant was removed, and 150 μL of dimethyl sulfoxide (DMSO) was added to dissolve the purple solid. The optical density value of each well was measured at 570 nm, and the percentage of cell viability in the biofilm was calculated from the following formula:

Cellviability(%)=[(Ap−Ab)/(Ac−Ab)]×100%

where Ap denotes the optical density (OD) value of the supernatant from the PC@P groups, Ab represents the OD value of the blank group well, and Ac indicates the OD value of the control group well.

**Activity against mature biofilms.** *S. aureus* USA300 and *S. aureus* USA300 LAC were cultured in Luria-Bertani (LB) medium at 37°C for 12 h, and then the bacterial suspension was diluted with Mueller Hinton (MH) medium to a cell density of 1 × 10^5^ CFU/mL as the working suspension. 100 μL of bacterial suspension was added to each well in a 96-well plate. The plate was incubated statically at 37°C for 24 h to obtain the mature biofilm, and then the old medium was removed. Fresh MH medium (100 μL) containing hydrogel extracts was added to corresponding wells for another 24 h incubation. After removing the culture solution, 100 μL of MTT solution (0.5 mg/mL) was added into each well and the plate was incubated for 4 h in the dark. The supernatant was removed, and 150 μL of dimethyl sulfoxide (DMSO) was added to dissolve the purple solid. The optical density value of each well was measured at 570 nm, and the percentage of cell viability in the biofilm was calculated from the following formula:

Cellviability(%)=[(Ap−Ab)/(Ac−Ab)]×100

where Ap denotes the optical density (OD) value of the supernatant from the PC@P groups, Ab represents the OD value of the blank group well, and Ac indicates the OD value of the control group well.

### 4.5 Cell culture

**Biocompatibility assessment.** To prepare the hydrogel extract, 1 mL of hydrogels was added to a 50 mL centrifuge tube, followed by the addition of 10 mL of DMEM medium, incubation for 3 days, and centrifugation (3000 rpm, 5 min) and extraction of the supernatant. NIH-3T3 cells were used for cell counting kit-8 (CCK-8) experiment and the live/dead staining assay. Cytocompatibility was evaluated using CCK-8. Briefly, NIH-3T3 cells (1 × 10^4^/mL) were seeded in 96-well plates treated with different hydrogel extracts and cultured for a specific time. The medium was removed after incubation, and a fresh culture medium containing 10% (v/v) CCK-8 was added to the 96-well plates, followed by incubation for 1 h at 37°C. The optical density at 450 nm was measured by a microplate reader. The live/dead staining assay (Calcein/PI Cell Viability Assay Kit) was performed to determine cell viability. The wells were washed three times with PBS, and then 100 μL of dye solution containing Calcein Acetoxymethyl Ester (Calcein AM, 1:1000) and propidium iodide (PI, 1:1000) was added to the wells in the incubator for 30 min. The images were photographed using a fluorescence microscope.

The hemolysis evaluation was conducted to evaluate the blood compatibility of materials. Whole blood samples were collected from male C57BL/6 J mice. Blood was centrifuged at 2000 rpm for 10 min, after which the RBCs were washed three times with phosphate-buffered saline (PBS). Subsequently, 5% (v/v) suspensions were prepared by diluting 200 μL of purified RBCs in 4 mL of PBS. Hydrogel-derived extracts (Gel, Gel-P@Z) were compared against negative (PBS) and positive controls (0.3% Triton X-100). 500 μL of solution from each experimental group was mixed with 500 μL of erythrocyte suspension in a 1.5 mL centrifuge tube. The tubes were incubated at 37°C for 1 h, followed by centrifugation at 3500 rpm for 10 min. All tubes were photographed and 200 μL of the supernatant was collected from each tube and its absorbance was measured at 540 nm.

**Anti-inflammatory effects evaluation.** To evaluate the effect of the hydrogel on driving macrophage phenotype polarization, RAW 264.7 or BMDMs were divided into five groups: (1) Negative control (Control): macrophages cultured in normal medium without LPS stimulation; (2) Positive group: macrophages stimulated with 100 ng/mL LPS; (3) Gel group: LPS-stimulated macrophages treated with Gel extraction solution; (4) Gel-P@Z group: LPS-stimulated macrophages treated with Gel-P@Z extraction solution. After 24 h of incubation, cells were harvested and analyzed by flow cytometry and immunofluorescence to assess macrophage polarization *in vitro*.

**Efferocytosis assay.** To induce apoptosis, HL60 cells were cultured in serum-free RPMI-1640 medium containing 30 μg/mL etoposide for 12 h in T25 flasks. The apoptotic cells were then harvested and fluorescently labeled by incubation with NHS-Fluorescein (5/6-carboxyfluorescein succinimidyl ester) in PBS for 30 min at rt in the dark. After three washes with PBS to remove excess dye, the prelabeled apoptotic HL60 cells were resuspended in BMDM-complete medium. These cells were added to BMDM monolayers in 12-well plates and co-cultured for 45 min to permit efferocytosis. Non-engulfed HL60 cells were removed by gentle washing with PBS. Finally, the BMDMs were collected and analyzed via flow cytometry and immunofluorescence to evaluate their efferocytic capacity.

**Cell migration assay.** Cell migratory capacity was assessed via both scratch wound healing and transwell assays. When the cells reached 90% confluence, a cell scratch was prepared using a 200 μL pipette tip, and the shed cells were washed with PBS three times. Then, normal medium or three hydrogel-derived extracts were added to the respective wells and cultured at 37°C. The scratch areas were calculated at 0 h (A_0_) and 24 h (A_t_), and the migration rate was calculated as follows:

$$\text{Migration}\;\text{rate}\;\left(\%\right)=\left(A_{0}-A_{t}\right)/A_{0}\times 100\%$$

For the transwell assay, 100 μL cell suspension (1 × 10^6^/mL) was added into the upper chamber of 12-well transwell plates and the lower chamber was filled with normal medium or the corresponding hydrogel extracts.

**Free-floating *in vitro* fascia culture.** Whole-back skin was harvested from euthanized mice using sterile scissors and forceps, with care taken to preserve the integrity of the underlying fascia. The samples were rinsed twice in ice-cold PBS to remove debris and placed epidermal-side down. The soft fascial layer was gently lifted with forceps, dissected into approximately 4-mm pieces, and washed again in PBS. These fascia explants were then transferred to 6-well plates containing either normal medium or medium supplemented with Gel or Gel-P@Z extracts. The medium was supplemented with additional extracts and refreshed during medium change. Fascia contraction was monitored daily by imaging under a stereomicroscope.

**Flow cytometry.** Qualified macrophages were collected, washed, and resuspended in cell staining buffer at a concentration of 10^7^/mL. Cells were blocked with anti-mouse CD16/32 antibody for 15 min and subsequently incubated with the following flow cytometry antibodies for 30 min: FITC-conjugated anti-CD86 antibody and APC-conjugated anti-F4/80 antibody. Quantitative analysis was performed on a flow cytometer, and both the mean fluorescence intensity (MFI) and the efferocytic ratio of macrophages were determined.

**Western Blot.** After treatment, cells in 6-well plates were lysed using RIPA buffer supplemented with a protease and phosphatase inhibitor cocktail. The lysates were centrifuged at 12,000 × g for 30 min at 4°C. Protein concentrations in the supernatants were determined using a bicinchoninic acid (BCA) assay kit. The proteins were mixed with 5 × loading buffer, denatured by boiling for 10 min, separated on 10% sodium dodecyl sulfate-polyacrylamide gels and transferred to 0.45-μm polyvinylidene fluoride membranes. After blocking with non-fat milk in TBST for 1 h at room temperature, membranes were incubated overnight at 4°C with specific primary antibodies. Following TBST washes, membranes were incubated with HRP-conjugated secondary antibodies for 1 h at room temperature, and were subsequently visualized using enhanced chemiluminescence.

### 4.6 Transcriptome sequencing (RNA-seq) and Bioinformatics analysis

To evaluate the effects on fibroblasts, NIH-3T3 cells were treated with either normal medium or Gel-P@Z extract for 24 h and total RNA was isolated using TRIzol reagent. Three replicates of each sample were sequenced. RNA purification, reverse transcription, library construction and sequencing were performed by Shanghai Majorbio Bio-pharm Biotechnology Co., Ltd. (Shanghai, China) according to the manufacturer's instruction (Illumina, USA). Briefly, eukaryotic mRNA was enriched with oligo (dT) magnetic beads and converted into cDNA libraries. Raw paired-end reads were quality-trimmed and filtered using fastp (v0.23.4) with default parameters. Clean reads were aligned to the reference genome in orientation-aware mode using HISAT2 (v2.2.1). Transcript assembly was performed with StringTie (v2.2.1) using a reference-guided approach. Differential expression analysis was performed using the R package DESeq2 (v1.46.0). Genes with |log_2_FC| ≥ 1 and FDR ≤0.05 were defined as significantly differentially expressed genes (DEGs). Functional enrichment analysis, including Gene Ontology (GO), Kyoto Encyclopedia of Genes and Genomes (KEGG) and Gene Set Enrichment Analysis (GSEA) were performed against the whole-transcriptome background using the clusterProfiler package (v4.14.6). Terms and pathways with a Benjamini-Hochberg-adjusted p-value ≤0.05 were considered significantly enriched.

### 4.7 Animal experiment

Male C57BL/6J mice (8 weeks of age) were used in the MRSA infected full-thickness wound model. Animals were deeply anesthetized with isoflurane prior to surgery. Two full-thickness skin injuries with a diameter of 6 mm were created on either side of the mice's backs using a biopsy punch. Immediately, 10 μL of *S. aureus* USA300 LAC suspension (1 × 10^6^ CFU/mL) was dropped to the wound sites, which were subsequently covered by Tegaderm dressing. After 24 h inoculation, wounds were treated with saline, Gel, Gel-P@Z. After an additional 24 h of infection, the mice were euthanized by intraperitoneal injection of pentobarbital sodium and the wound sites were excised, weighed, and homogenized in PBS containing 0.1% Triton X-100. The homogenate was plated on LB agar for CFU checking. The wound regions were imaged on day 0, 3, 7, 10 and 14 post-operation. The initial wound area was recorded as A_0_, and the wound area at each time point post-wounding was recorded as A_t_. Wound closure rate (%) was calculated using the following equation:

$$\text{Wound}\;\text{closure}\;\left(\%\right)=\left(A_{0}-A_{t}\right)\;/\;A_{0}\times 100\%$$

**Histological and immunofluorescence staining analysis.** The wound tissues were collected at day 3, 7, 10 and 14 and fixed with 4% paraformaldehyde solution. Then, the samples were dehydrated in an ethanol gradient, embedded in paraffin and cut into 5 μm-thick sections. A pathological section of the wound tissue was analyzed using Hematoxylin and eosin (H&E) and Masson's trichrome staining to observe the histological structure and collagen components of the wound samples. The histology images were obtained by a microscope.

In addition, immunofluorescence staining was conducted on the sections to reveal the progress during wound healing using the following primary antibodies: anti-F4/80, anti-CD86, anti-CD206, anti-Ki67, anti-CD31, anti-α-SMA, anti-Collagen I (Col I) and anti-Collagen III (Col III). Then the sections were incubated with species-matched Alexa Fluor-conjugated secondary antibodies. Finally, the sections were mounted with a DAPI-containing mounting solution and sealed with nail polish. Fluorescence images were captured using a fluorescence microscope.

### 4.8 Statistical analysis

Each experiment was independently repeated at least 3 times with similar results. All data were presented as mean ± SD and analyzed using GraphPad Prism 10.0 software (GraphPad, La Jolla, USA). Student's t-test was applied to compare two groups and one-way analysis of variance (ANOVA) was used among multi-group comparison. Data were considered statistically significant at p < 0.05 (*p < 0.05, **p < 0.01).

## Ethics approval and consent to participate

All animal experiments conducted in this study were performed in strict accordance with the National Institutes of Health Guide for the Care and Use of Laboratory Animals and approved by the Shanghai General Hospital Clinical Center Laboratory Animal Welfare and Ethics Committee (approval No. 2025AWS352).

## Funding

This work was supported by 10.13039/501100001809National Natural Science Foundation of China (22522504, 22305082), the Explorers Program of Shanghai (Grant No. 24TS1402900), the open research fund of Suzhou National Laboratory (No. SZLAB-1508-2025-TS035) and the Shanghai Youth Top Talent Program of Eastern Talent Plan (No. QNJY2025194).

## CRediT authorship contribution statement

**Chuanliang Fu:** Investigation, Writing – original draft. **Wenjing Zhang:** Conceptualization, Investigation, Writing – review & editing. **Renyuan Wang:** Investigation, Writing – review & editing. **Runyi Cao:** Investigation. **Zhengjie Chen:** Investigation. **Peilin Wang:** Investigation. **Ying Peng:** Investigation. **Yilin Huo:** Investigation. **Yi Hu:** Investigation. **Zun Ren:** Investigation. **Hao Zhang:** Investigation. **Yersen Mulat:** Investigation. **Zhengjie Luo:** Investigation. **Yahui Dai:** Investigation. **Min Zhou:** Formal analysis, Writing – review & editing. **Haodong Lin:** Conceptualization, Writing – review & editing.

## Declaration of competing interest

The authors declare that they have no known competing financial interests or personal relationship which could have appeared to influence the work reported in this paper.

## Data Availability

Data will be made available on request.
