# Supplementary material for: An infection-responsive multifunctional hydrogel enables potent activity against methicillin-resistant Staphylococcus aureus and promotes wound healing
Source: Mater Today Bio. 2026 Jul 25;39:103494. doi: 10.1016/j.mtbio.2026.103494 (PMC13445519; doi:10.1016/j.mtbio.2026.103494)
Supplement: Supplementary file 1 [file mmc1.docx]

**Supplemental Information**

**An Infection-Responsive Multifunctional Hydrogel Enables Potent Activity Against Methicillin-Resistant *Staphylococcus aureus* and Promotes Wound Healing**

Chuanliang Fu^a,1^, Wenjing Zhang^a,1,*^, Renyuan Wang^b,1^, Runyi Cao^b^, Zhengjie Chen^c^, Peilin Wang^a^, Ying Peng^a^, Yilin Huo^a^, Yi Hu^a^, Zun Ren^a^, Hao Zhang^a^, Yersen Mulat^a^, Zhengjie Luo^c^, Yahui Dai^a^, Min Zhou^c,*^, Haodong Lin^a,*^

^a^Trauma Center, Shanghai General Hospital, Shanghai Jiao Tong University School of Medicine, Shanghai 201620, China

^b^Precision Research Center for Refractory Diseases, Shanghai General Hospital, Shanghai Jiao Tong University School of Medicine, Shanghai 201620, China

^c^Key Laboratory for Ultrafine Materials of Ministry of Education, Frontiers Science Center for Materiobiology and Dynamic Chemistry, Shanghai Frontiers Science Center of Optogenetic Techniques for Cell Metabolism, Research Center for Biomedical Materials of Ministry of Education, School of Materials Science and Engineering, East China University of Science and Technology, Shanghai 200237, China

^*^Corresponding authors.

*E-mail addresses:* 72400019193@shsmu.edu.cn (H. Lin), zhangwenjing3232@163.com (W. Zhang), [minzhou@ecust.edu.cn](mailto:rliu@ecust.edu.cn) (M. Zhou)

^1^These authors contributed equally to this work.

**1. Materials and methods**

**1.1 Materials**

The solvent of anhydrous tetrahydrofuran (THF), anhydrous dichloromethane (DCM), anhydrous N,N-Dimethylformamide (DMF) and anhydrous N,N-dimethylacetamide (DMAc), methyl trifluoromethanesulfonate (MeOTf) and N-Boc-glycine, biopsy punch (WHAWB100040) were purchased from Sigma-Aldrich and used without further purification. The rest chemical reagents and solvents were purchased from Adamas-beta and used without further purification. Penicillin–streptomycin purchased from NCM Biotech in China. Etoposide (HY-13629) was purchased from MCE. NHS-FLUORESCEIN (TAMRA-NHS, 46410), fetal bovine serum (FBS) (10100147), TRIzol reagent (15596026CN) were purchased from Invitrogen. Dulbecco's modified Eagle's medium (DMEM, C11995500BT), RPMI-1640 (C11875500BT) were purchased from Gibco. Cell counting kit-8 (CCK-8, A311-02) was purchased from Vazyme. The live/dead staining assay (Calcein/PI Cell Viability Assay Kit, C2015S), RIPA buffer (P0038), bicinchoninic acid (BCA) assay kit (P0011), 5 × loading buffer (P0015) were purchased from Beyotime Biotechnology. Hematoxylin-Eosin (H&E) (G1120) and Masson’s trichrome (G1346) staining were purchased from Solarbio. Protease and phosphatase inhibitor cocktail (78442), SuperSignal West Pico PLUS Chemiluminescent Substrate (34580) were purchased from ThermoFisher Scientific. 0.45-μm polyvinylidene fluoride membranes (IPVH00010) were purchased from Millipore. 12-well transwell plates (3422) were purchased from Corning. M-CSF were purchased from Peprotech. Rabbit anti-iNOS (13120T), rabbit anti-CD86 (19589T), rabbit anti-Phospho-SMAD2/3 (8828T) and mouse anti-α-SMA (48938T) were purchased from CST. Rabbit anti-ARG1 (A1847) was purchased from Abclonal. Mouse anti-Beta Actin (66009-1-Ig), rabbit anti-Col I (14695-1-AP), rabbit anti-COL III (22734-1-AP) and rabbit anti-Vinculin (26520-1-AP) were purchased from Proteintech. Rabbit anti-F4/80 (GB113373-100) was purchased from Servicebio. Mouse anti-CD206 (HA601453) and rabbit anti-Smad2/3 (HA724174) were purchased from HUABIO. Mouse anti-Ki67 (550609) was purchased from BD Pharmingen. Rabbit anti-CD31 (ab28364) was purchased from Abcam. Mouse anti-TGFβ1 (BF8012) was purchased from Affinity. Peroxidase AffiniPure^TM^ Goat Anti-Mouse IgG (H + L) (115-035-003) and Peroxidase AffiniPure^TM^ Goat Anti-Rabbit IgG (H + L) (111-035-144) were purchased from THE JACKSON LABORATORY. Cell staining buffer (420201), anti-mouse CD16/32 (156604), FITC-conjugated anti-CD86 antibody (159219) and APC-conjugated anti-F4/80 antibody (123116) were purchased from Biolegend. NIH-3T3 (ATCC CRL-1658), RAW264.7 (ATCC TIB-71) and HL60 (ATCC CCL-240) cell lines were purchased from the Cell Bank of the Chinese Academy of Sciences (Shanghai, China). *Staphylococcus aureus* (methicillin-resistant *S. aureus* USA300), *Staphylococcus aureus* (methicillin-resistant *S. aureus* USA300 LAC) are clinical isolated strains which were provided by Shanghai Ruijin Rehabilitation Hospital.

**1.2 Instrument and characterization**

Synthesized chemicals were purified using a SepaBean machine equipped with Sepaflash columns produced by Santai Technologies Inc. in China. Gel permeation chromatography (GPC) characterization was performed on a Waters GPC instrument equipped with a Waters 1515 isocratic HPLC pump and a Waters 2414 refractive index detector. The GPC were equipped with a Tosoh TSKgel Alpha-2500 column (particle size 7 µm) and a Tosoh TSKgel Alpha-3000 column (particle size 7 µm) linked in series. Relative number-average molecular weight, degree of polymerization (DP) and dispersity index (Ð) were calculated from a calibration curve using polymethylmethacrylate (PMMA) as standards. ^1^H nuclear magnetic resonance (NMR) spectra were collected on a Varian MercuryPlus 600 spectrometer at 600 MHz using D_2_O as the solvent. The surface morphology of Gel scaffolds was observed on a S-4800 Field Emission Scanning Electron Microscope (FESEM). The morphology of bacteria was analyzed using FESEM (JEOL JSM-IT800).Measurements for size distribution and zeta potential were conducted on a Malvern Zetasizer Nano ZSZEN3700 dynamic light scattering (DLS) nanoparticle analyzer. The rheological properties of the scaffolds were measured using rheometer (Haake Mars40, Thermo Fisher Scientific). The fluorescence intensity of fluorescamine was recorded on a plate reader (SPECTRAmax 384, Molecular Devices). FT-IR spectra were collected on attenuated total reflection FT-IR spectrometer (INVENIO, Bruker). Thermogravimetric analysis (TGA) was performed using a TGA 8000 (Perkin Elmer). The structural property of nanoparticles was analyzed using XRD (Rigaku Smart Lab 3 kW).

**1.3 Rheological properties.**

Rheological properties of Gel hydrogel was measured by a stress-controlled rheometer equipped with a 25 mm plate-plate. The rheometer testing process involves equilibrating on the instrument for 30 s, followed by 1 min of UV exposure, and then a final 5-min equilibration period after the UV exposure ends. For this studies, storage modulus (elastic modulus, G’) and loss modulus (viscous modulus, G”) were both analyzed. All the tests were performed at 50 ℃. For oscillatory time sweep experiments, the constant strain and frequency were fixed at 10 % and 1 Hz, respectively. The gelation time was determined as the time when G’ became higher than G”.

**1.4 Cell culture**

NIH-3T3 and Raw264.7 cells were cultured in high-glucose Dulbecco's Modified Eagle Medium (DMEM) with 10% fetal bovine serum (FBS) and 1% penicillin–streptomycin. HL60 cells were maintained in RPMI-1640 medium with 10% FBS. Bone marrow-derived macrophages (BMDMs) were isolated from 5-week-old male C57BL/6 mice euthanized by cervical dislocation. Following surface sterilization in 75% ethanol for 3 minutes, femurs and tibiae were aseptically dissected. Bone ends were cut with sterile scissors, and the marrow cavities were flushed using complete medium. The cell suspension was centrifuged at 300 × g for 3 min. The pelleted cells were then resuspended and cultured in complete medium containing 20 ng/mL recombinant M-CSF at a density of 1 × 10⁷ cells/mL in 100-mm polystyrene culture dishes. Medium was replaced every other day. After 7 days of differentiation, BMDMs were harvested and reseeded at appropriate densities for subsequent assays. All cells were maintained at 37°C in a humidified 5% CO₂ atmosphere.

**Quantitative real-time PCR.** Total RNA was isolated from the treated cells using TRIzol reagent according to the manufacturer’s instructions. Subsequently, 1,000 ng of total RNA was reverse-transcribed into complementary DNA (cDNA) using the HiScript III RT SuperMix. Quantitative real-time polymerase chain reaction (qRT-PCR) was performed using a Roche LightCycler 480 II (Roche, Switzerland). Primer sequences are listed in Table S1. Gene expression was normalized to Gapdh and analyzed via the 2^(-ΔΔCt) method.

**2. Supporting figure**

Supplement Fig. 1. ^1^H NMR spectrum of Gly-POX as a TFA salt.

Supplement Fig. 2. Drug loading properties of different mass ratio of Gly-POX to ZIF-8 (n=3).Data are presented as mean ± SD. *p < 0.05, **p < 0.01; ns, not significant.

Supplement Fig. 3. Zeta potentials of ZIF-8 and Gly-POX@ZIF-8 determined by DLS. Data are presented as mean ± SD. *p < 0.05, **p < 0.01; ns, not significant.

Supplement Fig. 4. Rheological time sweep of Gel and Gel-P@Z by measuring the storage modulus (G′) and loss modulus (G″) of the hydrogel at a fixed strain (10%) and frequency (1 Hz).

Supplement Fig. 5. Inhibitory effect of Gel and Gel-P@Z against MRSA biofilm formation (left). Ability of Gel and Gel-P@Z to eradicate mature MRSA biofilm (right).

Supplement Fig. 6. Inhibitory effect of Gel and Gel-P@Z against MRSA biofilm formation (left, n = 3). Ability of Gel and Gel-P@Z to eradicate mature MRSA biofilm (right, n = 3).

Supplement Fig. 7. Changes in morphology of BMDMs after treatment.

Supplement Fig.8. Immunofluorescence images of iNOS, Arg-1 and F4/80 staining of BMDMs.

Supplement Fig. 9. Relative *Tnf*, *Il6*, *Il10* and *Il4* mRNA expression of BMDMs after treatment (n = 4).

Supplement Fig. 10. Relative *Col1a1* and *Acta2* mRNA expression of NIH-3T3 after treatment (n = 4).

**3. Table**

Table S1. Primer sequences used for qRT-PCR

| Gene | Primer | Sequence (5’ - 3’) |
| --- | --- | --- |
| *Gapdh* | Forward | TGATGGGTGTGAACCACGAG |
|  | Reverse | AGTGATGGCATGGACTGTGG |
| *Tnf* | Forward | ATGGCCTCCCTCTCATCAGT |
|  | Reverse | AAGGTACAACCCATCGGCTG |
| *Il6* | Forward | GCCTTCTTGGGACTGATGCT |
|  | Reverse | AGCCTCCGACTTGTGAAGTG |
| *Il10* | Forward | CAGAGAAGCATGGCCCAGAA |
|  | Reverse | GCTCCACTGCCTTGCTCTTA |
| *Il4* | Forward | CCATATCCACGGATGCGACA |
|  | Reverse | AGGACGTTTGGCACATCCAT |
| *Col1a1* | Forward | CCCAGTGGCGGTTATGACTT |
|  | Reverse | CTCAAGGTCACGGTCACGAA |
| *Acta2* | Forward | GGCATCCACGAAACCACCTA |
|  | Reverse | AATGCCTGGGTACATGGTGG |
